# Small-molecule activation of SERCA2a SUMOylation for the treatment of heart failure

**DOI:** 10.1038/ncomms8229

**Published:** 2015-06-12

**Authors:** Changwon Kho, Ahyoung Lee, Dongtak Jeong, Jae Gyun Oh, Przemek A. Gorski, Kenneth Fish, Roberto Sanchez, Robert J. DeVita, Geir Christensen, Russell Dahl, Roger J. Hajjar

**Affiliations:** 1 Department of Medicine/Cardiology, Cardiovascular Research Center, Icahn School of Medicine at Mount Sinai, 1 Gustave L. Levy place, Box 1030, New York, New York 10029, USA; 2 Department of Structural and Chemical Biology, Icahn School of Medicine at Mount Sinai, New York, New York 10029, USA; 3 Department of Pharmacology and System Therapeutics, Experimental Therapeutics Institute, Icahn School of Medicine at Mount Sinai, New York, New York 10029, USA; 4 Institute for Experimental Medical Research, Oslo University Hospital Ullevål and University of Oslo, Oslo 0450, Norway; 5 Department of Pharmaceutical Science, Rosalind Franklin University of Medicine and Science, North Chicago, Illinois 60064, USA

## Abstract

Decreased activity and expression of the cardiac sarcoplasmic reticulum calcium ATPase (SERCA2a), a critical pump regulating calcium cycling in cardiomyocyte, are hallmarks of heart failure. We have previously described a role for the small ubiquitin-like modifier type 1 (SUMO-1) as a regulator of SERCA2a and have shown that gene transfer of SUMO-1 in rodents and large animal models of heart failure restores cardiac function. Here, we identify and characterize a small molecule, N106, which increases SUMOylation of SERCA2a. This compound directly activates the SUMO-activating enzyme, E1 ligase, and triggers intrinsic SUMOylation of SERCA2a. We identify a pocket on SUMO E1 likely to be responsible for N106’s effect. N106 treatment increases contractile properties of cultured rat cardiomyocytes and significantly improves ventricular function in mice with heart failure. This first-in-class small-molecule activator targeting SERCA2a SUMOylation may serve as a potential therapeutic strategy for treatment of heart failure.

## Abstract (web summary)

SUMOylation of the cardiac calcium pump SERCA2a affects its activity and promotes cardiomyocyte contractility. Here the authors identify a small molecule N106 that increases SERCA2 SUMOylation and improves heart function in mice, and propose a promising therapeutic strategy for treatment of heart failure.

## Graphical abstract

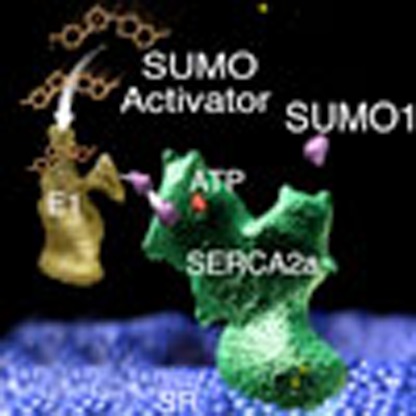

There are 26 million people who suffer from heart failure (HF) in the world (5.8 million patients in the United States)^1^. Of all the cardiovascular diseases, HF is the only diagnosis increasing in both incidence and prevalence^2^. For this reason, there is a critical need for novel targets and treatment strategies. The cardiac sarcoplasmic reticulum Ca^2+^-ATPase (SERCA2a) is a key pump responsible for intracellular calcium handling and contractility in cardiac cells. Impaired calcium reuptake resulting from decreased expression and activity of SERCA2a is a hallmark of HF. The work in our laboratory has led to the successful completion of Phase 1 and Phase 2 clinical trials of adeno-associated vector type 1 (AAV1)-mediated gene transfer of SERCA2a in patients with severe HF, showing clinical benefits in patients receiving AAV1.SERCA2a^3^,^4^,^5^,^6^. An international Phase 2b/3 trial of 250 patients has recently completed enrolment. Beyond its effects on enhancing contractility, SERCA2a gene transfer has been shown to restore cardiac energetics^7^,^8^, decrease ventricular arrhythmias^9^,^10^, block smooth muscle cell proliferation and enhance coronary flow through the activation of nitric oxide synthase in endothelial cells^11^. We recently found that in HF, there are changes of posttranslational modifications (PTMs) of SERCA2a that render it dysfunctional and we showed that restoration of SERCA2a by gene transfer does not abrogate the PTMs of the transporter. Therefore, SERCA2a’s enzymatic dysfunction, in addition to its decreased expression, needs to be addressed in the failing heart, to normalize calcium cycling.

SUMOylation, a ubiquitin-like reversible PTM where SUMO (small ubiquitin-like modifier) covalently attaches to a target protein through specific enzyme cascades reaction, has been shown to be implicated in controlling many cellular processes, including regulation of protein function, stability and localization^12^. Several studies have demonstrated that protein SUMOylation is associated with critical cellular pathways, many of them ultimately affecting cardiac function and development, suggesting SUMOylation as a valuable target for the treatment of cardiovascular diseases^13^,^14^. We have recently found that the levels and activity of SERCA2a in cardiomyocytes to be modulated in parallel with the levels of small ubiquitin-like modifier type 1 (SUMO-1). SUMO-1 gene transfer led to restoration of SERCA2a levels, improved haemodynamic performance and reduced mortality in a murine model of pressure overload-induced HF^15^. We further demonstrated that gene transfer of SUMO-1 in combination with SERCA2a led to reversal of HF in a porcine model of ischaemic HF^16^.

In this report, we have conducted a screening study to discover small molecules hat increase SUMOylation. We identify a small molecule, N106 (*N*-(4-methoxybenzo[d]thiazol-2-yl)-5-(4-methoxyphenyl)-1,3,4-oxadiazol-2-amine (molecular weight 354gpermol)), that enhances SERCA2a SUMOylation, resulting in enhanced contractility both *in vitro* and *in vivo*. In addition, we characterize the putative site on E1 ligase where N106 docks and confers its activity. Thus, our studies have established a compelling rationale for targeting SUMOylation of SERCA2a as a new therapeutic approach for the treatment of HF.

## Results

### Identification of potent SERCA2a SUMOylation activators

To identify small-molecule activators of the SUMOylation pathway, we used the data from *in-vitro* SUMOylation of Ran GTPase-activating protein 1 (RanGAP1, a well-known substrate of SUMO-1)^17^. To find novel small molecules capable of activating SUMOylation, an AlphaScreen (PerkinElmer) assay was developed to detect SUMOylation of RanGAP1 with SUMO-1. AlphaScreen is a bead-based fluorescence resonance energy transfer (FRET) technology that results in emission of light when donor and acceptor beads are brought into proximity. A His-tagged RanGAP1 was used as the substrate for the addition of a glutathione *S*-transferase (GST)-tagged form of SUMO-1 in the presence of E1, E2 (Ubc9) and ATP. Following the SUMOylation reaction, glutathione-coated donor beads and Ni^2+^-coated acceptor beads are added to interact with GST and His, respectively, effectively capturing the SUMOylated substrate. When the beads are brought into proximity by the SUMOylated protein, irradiation of the donor beads at 680 nm will result in emission of light at 520–620 nm. The *K*_d_ determined by FRET between SUMO-1 and RanGAP1 is compatible with those determined with other traditional approaches, such as isothermal titration calorimetry and surface plasmon resonance. We have used this technology to screen over 100,000 compounds from large NCI libraries and a few compounds have been shown to activate SUMOylation at a concentration of 10 μM. Among molecules that increased the RanGAP1 SUMOylation, we performed subsequent functional assays. A compound was considered a primary hit if it increased the FRET signal greater than threefold the s.d. above the mean of the vehicle controls when tested at 10 μM. Hits were subsequently filtered to remove all compounds containing substructural features common to promiscuous compounds^18^. Compounds that also appeared as frequent hits in other FRET-based assays were also excluded. We began by screening 50 small molecules for their ability to SUMOylate SERCA2a in HEK-293 cells at 10 μM concentration, 14 of which were non-toxic. Of the 14 compounds tested, we selected eight compounds that increased SERCA2a SUMOylation in HEK-293 cells overexpressing SERCA2a (Supplementary Fig. 1a). We then assessed the mechanical properties of the eight compounds using a video-based edge-detection system in isolated cardiomyocytes (Supplementary Fig. 1b,c). Endogenous SERCA2a SUMOylation status was also determined in the same set of cells (Supplementary Fig. 1d). In this study, we focused on the small molecules that activate SERCA2a SUMOylation and that improve cardiac function. Of them, only two compounds (N6 and N106) showed both positive inotropic effects and enhancement of endogenous SERCA2a SUMOylation without upregulation of Ubc9 (Supplementary Fig. 1e,f).

### Activation of cardiomyocyte mechanical properties by N106

**Figure 1:**
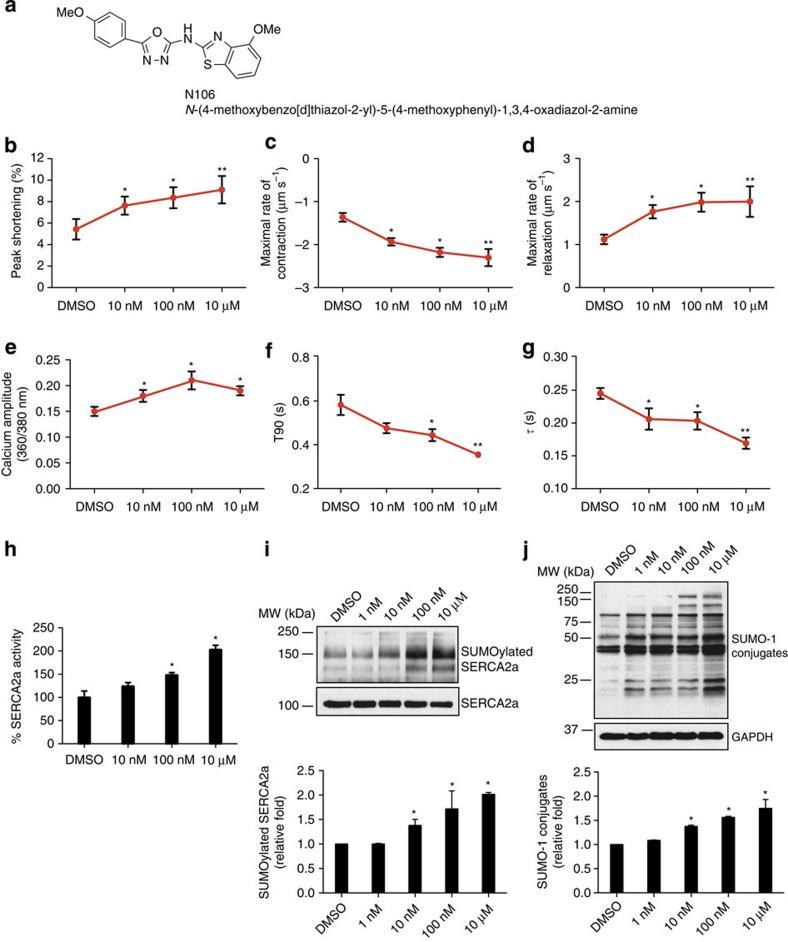
N106 increases SERCA2a’s cellular activity and SUMOylation in adult cardiomyocytes. *In-vitro* characterization of activators of SERCA2a SUMOylation. Cell-based screening of small molecules identified one promising compound. Adult ventricular cardiomyocytes were isolated from Sprague–Dawley rats and treated with small molecules on dose-dependent manner for 24 h. DMSO-treated cardiomyocytes were used as a negative control. (**a**) Chemical structure of SERCA2a SUMOylation activator. N106, *N*-(4-methoxybenzo[d]thiazol-2-yl)-5-(4-methoxyphenyl)-1,3,4-oxadiazol-2-amine. (**b-g**) The effects of N106 compound in cardiomyocyte contractility and calcium transient. The dose-dependent effects of N106 compound presented on peak shortening, the time to maximal departure velocity, the time to maximal return velocity, calcium ratio, decay time constant (*τ*) and the time to 90% baseline (t90) fluorescence in cardiomyocytes were assessed using a video-based edge-detection system (IonOptix Inc.). *n*=25 cardiomyocytes from each three of hearts per condition. (**h**) The effects of N106 compound on the ATPase activity of SERCA2a. The ATPase activity were measured at 25 °C in 50 mM MOPS, 100 mM KCl, 5 mM MgCl_2_, 1 mM EDTA at pCa 5.0 by using a coupled enzyme system consisting of pyruvate kinase and lactate dehydrogenase, and monitoring the oxidation of the reduced form of NADH at 340 nM. The enzyme activity was determined by using same set of cardiomyocytes. (**i**) The effect of N106 compound for the formation of SUMOylated SERCA2a was determined using the same set of cardiomyocytes. Endogenous SERCA2a SUMOylation was detected by immunoprecipitation with anti-SERCA2a and then western blotting with anti-SUMO-1 antibodies. The experiments shown are representative of three independent experiments with similar results. (**j**) The effect of N106 compound on global SUMOylation was measured by western blot analysis with an anti-SUMO-1 antibody in the same set of cardiomyocytes. The experiments shown are representative of three independent western blottings. Data are represented as mean±s.e.m. (*n*=3 mice hearts per each condition). Data are represented as mean±s.e.m. *P≤0.05; **P<0.001 (Student’s *t*-test) from DMSO control.

Of all the compounds studied, N106 had the most pronounced cellular and molecular effects. Treatment with this compound significantly increased cell contraction (peak shortening (%); N106 12.03±0.80, dimethylsulfoxide (DMSO) 3.05±0.47; *P*<0.001, Student’s *t*-test, *n*=25 cells per heart, three hearts were used) and reduced the rate of transient calcium decay (*τ* (s); N106 0.18±0.02, DMSO 0.24±0.02; *P*<0.05, Student’s *t*-test), and significantly induced endogenous SERCA2a SUMOylation in isolated adult cardiomyocytes (Supplementary Fig. 1b–f). The chemical structure of N106 compound is shown in Fig. 1a. To further validate the efficacy of N106 for cardiac function, we performed dose–response experiments. The results indicated that N106 significantly improves cell contractility (peak shortening (%): 10 nM, 7.63±0.48, *P*<0.05; 100 nM, 8.36±0.56, *P*<0.05; and 10 μM, 9.10±0.52, *P*<0.001, Student’s *t*-test; maximal velocities of cell contraction (μm s^−1^): 10 nM, −1.93±0.08, *P*<0.05, Student’s *t*-test; 100 nM, −2.17±0.10, *P*<0.05, Student’s *t*-test; and 10 μM, −2.30±0.19, *P*<0.001, Student’s *t*-test); and relaxation (10 nM, 1.76±0.08, *P*<0.05, Student’s *t*-test; 100 nM, 1.98±0.12, *P*<0.05, Student’s *t*-test; and 10 μM, 1.99±0.15, *P*<0.001, Student’s *t*-test) in a concentration-dependent manner (Fig. 1b-d). Calcium-transient amplitude was significantly increased following N106 treatment (10 nM, 0.18±0.01, *P*<0.05, Student’s *t*-test; 100 nM, 0.21±0.01, *P*<0.05, Student’s *t*-test; and 10 μM, 0.19±0.00, *P*<0.05, Student’s *t*-test; Fig. 1e). Time constant for relaxation (time for 90% decay, t90 (s): 10 nM, 0.47±0.02, *P*<0.05, Student’s *t*-test; 100 nM, 0.44±0.02, *P*<0.05, Student’s *t*-test; and 10 μM, 0.35±0.006, *P*<0.001, Student’s *t*-test; exponential decay of time constant, *τ* (s): 10 nM, 0.20±0.01, *P*<0.05, Student’s *t*-test; 100 nM, 0.20±0.01, *P*<0.05, Student’s *t*-test; and 10 μM, 0.16±0.008, *P*<0.001, Student’s *t*-test) was also significantly decreased with increasing amounts of N106, indicating faster SERCA2a-mediated Ca^2+^ uptake (Fig. 1f,g). Furthermore, increasing N106 concentration increased SERCA2a ATPase activity (Fig. 1h) and levels of SERCA2a SUMOylation (Fig. 1i). Whole SUMO-1 conjugates were increased in response to the concentration of N106 (Fig. 1j). These data indicate that N106 enhances SERCA2a SUMOylation and its cellular activity in a concentration-dependent manner.

**Figure 2:**
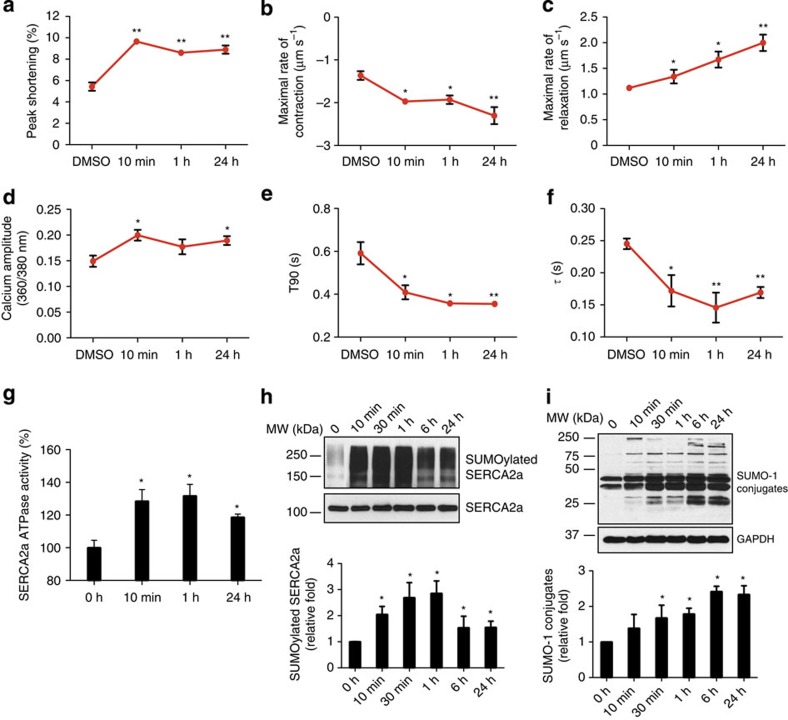
Time-course experiments with N106 in isolated adult cardiomyocytes. (**a–f**) Adult ventricular cardiomyocytes were isolated from Sprague–Dawley rats and treated with 10 μM of N106 compound over a time course as indicated. The time course of the contractile response to N106 was determined as peak shortening (**a**), the time to maximal velocity of sarcomere shortening (**b**) and re-lengthening (**c**). The time-course changes in calcium kinetic parameters such as calcium transient (**d**), decay time constant (**e**) and the time to 90% baseline fluorescence in cardiomyocytes (**f**) were detected. *n*=25 cardiomyocytes from each three of hearts per condition. DMSO was used as vehicle control. The time courses of the ATPase activity of SERCA2a (**g**), level of SUMOylated SERCA2a (**h**) and level of SUMO-1 conjugates (**i**) were determined by using the same set of cardiomyocytes (two independent experiments). Data are represented as mean±s.e.m. *P≤0.05; **P<0.001 (Student’s *t*-test) from DMSO control.

Time-course experiments showed that N106 increased cell contractility (peak shortening (%): 10 min, 9.65±0.27, *P*<0.001, Student’s *t*-test; 1 h, 8.60±0.23, *P*<0.001, Student’s *t*-test; and 24 h, 8.90±0.38, *P*<0.001, Student’s *t*-test; maximal velocities of cell contraction (μm s^−1^): 10 min, −1.97±0.04, *P*<0.05, Student’s *t*-test;1 h, −1.92±0.09, *P*<0.05, Student’s *t*-test; and 24 h, −2.3±0.19, *P*<0.001, Student’s *t*-test; maximal velocities of cell relaxation (μm s^−1^): 10 min, 1.12±0.11, *P*<0.05, Student’s *t*-test; 1 h, 1.67±0.15, *P*<0.05, Student’s *t*-test; and 24 h, 1.99±0.15, *P*<0.001, Student’s *t*-test; calcium transient (calcium amplitude (ratio 360/380 nm): 10 min, 0.19±0.01, *P*<0.05, Student’s *t*-test; 1 h, 0.17±0.01, *P*=0.17, Student’s *t*-test; 24 h, 0.18±0.008, *P*<0.05, Student’s *t*-test; t90 (s): 10 min, 0.40±0.03, *P*<0.05, Student’s *t*-test; 1 h, 0.35±0.01, *P*<0.05, Student’s *t*-test; and 24 h, 0.35±0.006, *P*<0.001, Student’s *t*-test; *τ* (s): 10 min, 0.17±0.02, *P*<0.05, Student’s *t*-test; 1 h, 0.14±0.02, *P*<0.001, Student’s *t*-test; and 24 h, 0.16±0.008, *P*<0.001, Student’s *t*-test), SERCA2a’s ATPase activity and SUMOylation short amount of time (10∼30 min), and these effects were sustained at 24 h in adult cardiomyocytes (Fig. 2).

### Mechanism of action by N106 through SUMO E1 enzyme

**Figure 3:**
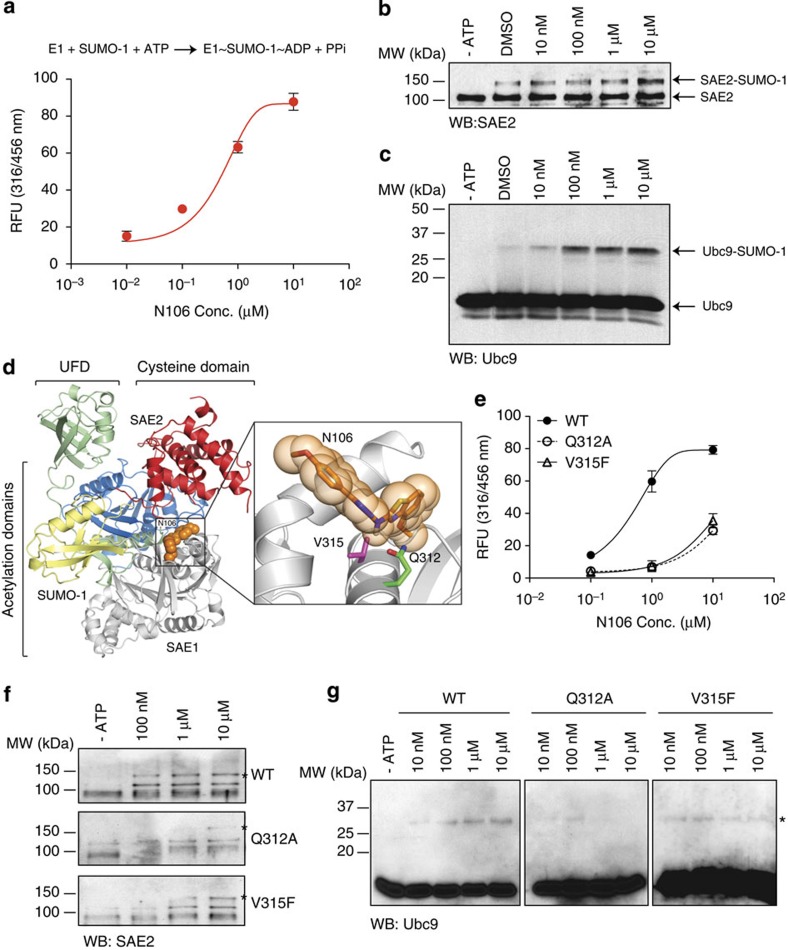
SUMO-specific activating enzyme, E1, is a direct target of N106. Mechanism of action of N106 on individual steps in the SUMOylation cascade *in vitro*. **(a)** Inorganic pyrophosphate resulting from ATP hydrolysis in E1-catalysed SUMO-1 activation in the presence of increasing concentration of N106 compound was assessed by fluorogenic pyrophosphate assay kit. Recombinant human SUMO-specific E1 (0.05 μM), human Ubc9 (0.25 μM), human SUMO-1 (12.5 μM) and ATP (1 mM) were incubated with increasing concentration of N106 for 30 min at 37 °C and quantified using a fluorescence microplate reader. (**b**) The SAE2-SUMO-1 conjugation was detected followed by western blotting with anti-SAE2 antibodies. SUMO E1 (0.05 μM), SUMO-1 (12.5 μM) and ATP (1 mM) were co-incubated with increasing concentration of N106 for 30 min at 37 °C. The reaction mixtures were separated on 7.5% SDS–PAGE gel under non-reducing conditions. **(c)** E2-SUMO-1 conjugation was visualized by western blotting with anti-Ubc9 antibody. SUMO E1 (0.05 μM), Ubc9 (0.25 μM), SUMO-1 (12.5 μM) and ATP (1 mM) were incubated without or with increasing concentration of N106 for 30 min at 37 °C. SUMO-specific E1-dependent E2-SUMO-1 conjugation was visualized by western blotting with anti-Ubc9 antibodies. (**d**) Ribbon representation for the SUMO E1/SUMO-1-N106. Atoms for the N106, SUMO-1 (yellow) and SUMO E1 domains are shown (SAE1 in grey and SAE2 consists of three domains such as ubiquitin-folding domain (UFD) in green, adenylation domain in blue and cysteine domain in red). The structure of the human SUMO-activating enzyme, PDB Code 3kyd was used as a model. Residues target mutagenesis in putative binding pocket for N106 to SUMO-activating enzyme are shown in close-up images (glutamine 312 in green and valine 315 in purple). Alternations in the putative binding pocket decrease the N106-mediated biochemical activity of SUMO E1. (**e**) ATP hydrolytic activity of WT and mutant SUMO E1 are determined. (**f**) The E1-SUMO-1 conjugation was detected followed by western blot analysis with anti-SAE2 antibody. *The SAE2-SUMO-1 conjugated bands. **(g)** The E1-dependent Ubc9-SUMO-1 conjugation was visualized by western blotting with anti-Ubc9 antibody. *The E2-SUMO1-conjugating bands. Three independent experiments were performed. Data are represented as mean±s.e.m. WB, western blotting.

To understand the mechanisms of action by which N106 enhances SERCA2a SUMOylation, we tested the effects of N106 on SUMO E1 activity. We decided to focus on the SUMO E1 activity, as only SUMO E1 and SUMO E2 enzymes were used in the initial screen. Furthermore, the SUMOylation pathway uses a single SUMO E1 enzyme and SUMO E1 is the first control point of SUMOylation cascade. The SUMO E1 activity was determined by three independent experiments. N106 stimulated ATP-dependent activation of SUMO E1 (EC_50_=2.45±0.13 μM, Fig. 3a), thioester formation between the SUMO-1 and SUMO E1 (Fig. 3b), and subsequent transfer of the activated SUMO-1 from SUMO E1 to the unique SUMO E2 enzyme, Ubc9, in a dose-dependent manner (Fig. 3c). The SUMO conjugation was not observed in the presence of reducing agent, β-mercaptoethanol, indicating a thioester bond (Supplementary Fig. 2a,b). We further asked whether there was a dose-limiting effect. The maximal tolerated doses of N106 for *in-vitro* thioester formation between the SUMO E1 and SUMO-1 was ∼20 μM range. The SUMO-1 conjugation to SUMO E1 enzyme and SUMO E1-dependent thioester bond formation between SUMO E2 enzyme and SUMO-1 were less pronounced at 50 μM concentration and that was inhibited at over 100 μM concentration (Supplementary Fig. 3a,b). In addition, the positive effect of N106 on cardiomyocytes contractility was no longer observed at over 50 μM concentration of N106 (Supplementary Fig. 3c). Taken together, all three experiments suggest that SUMO E1 is a direct target of N106.

To better understand the structural basis for the action of N106 on SUMO E1, we performed computational modelling studies. As SUMO E1 undergoes massive conformational changes as it transitions between the open (the resting state) and closed (the activating state) states during the SUMOylation process^19^, we used structures that represent both states as modelling templates. Interestingly, we found that a promising binding pocket for N106 is located at the interface between subunit 1 (SAE1) and subunit 2 (SAE2) that only exists in the closed state of SUMO E1 and not in the open-state structure (Fig. 3d). There are two relevant residues in this pocket, valine 315 (V315) and glutamine 312 (Q312) on SAE1. According to the docking model, V 315 (in purple) points into the N106 binding site; hence, we decided to mutate it to a larger residue such as phenylalanine (V315F). The docking solution also suggests that Q312 (in green) establishes hydrogen bonds with the N106 compound. In this case, we mutated Q312 to alanine (Q312A), to remove the capacity for hydrogen-bond formation. To validate the putative binding site of SAE1 on the pocket that exist in the closed state of SUMO E1, we tested SUMO E1 activity using these mutants of SAE1 with N106. The Q312A and V315F mutants of putative N106 binding site showed significantly lower ATP hydrolysis activity as compared with the wild-type (WT) protein (Fig. 3e). Moreover, on ATP-dependent SUMO E1 activation in the presence of N106, SUMO E1-SUMO and SUMO E2-SUMO conjugations were weaker in the mutant SAE1 as compared with the WT protein (Fig. 3f,g). Finally, Q312A mutant showed the lowest ATP hydrolysis, SUMO conjugation and thioester transfer to the E2, which may suggest the importance of Q312 residue in SUMO E1 for N106-mediated activation. Moreover, N106-induced increase in SUMOylation of SERCA2a was weaker in mutant SAE1-overexpressing cells than in overexpressed WT (Supplementary Fig. 4). Dose-dependent increases in the profiles of SERCA2a SUMOylation induced by N106 was not observed in cells overexpressing mutants (Supplementary Fig. 4). These data suggest that both the Q312 and V315 residues on SAE1 could be the potential N106-binding sites.

Importantly, N106 did not significantly stimulate any *in-vitro* activity of the ubiquitin-activating enzyme (Ub E1) at concentrations up to 100 μM, including ubiquitin activation, conjugation of ubiquitin to Ub E1 enzyme and Ub E1-dependent conjugation of ubiquitin to the E2 enzyme cdc32 (Supplementary Fig. 5). It is important to note that the N106-binding pockets are not present in the Ub E1 enzyme, further indicating that the N106 compound is specific for SUMO E1 enzyme. It is also well known that each ubiquitin-like modifier (for example, ubiquitin, SUMO, NEDD8 and so on) has its own specific activating E1 enzyme that serves as the entry point for an enzyme cascade^20^. Thus, our screen identified a novel activator of the SUMO E1 enzyme.

### Haemodynamic effects of N106 in mouse model of HF

On the basis of our biochemical and computational studies, and given the findings obtained in cardiomyocytes, N106 should increase cardiac function via SERCA2a SUMOylation activation in an animal model of HF. To test our hypothesis, we measured haemodynamic parameters to evaluate the acute effects of N106 infusion on the cardiac function of the mouse model of HF. In the first set of experiments, mice were exposed to pressure overload by transverse aortic constriction (TAC) or sham operation and received either N106 or vehicle. At 2 months, post TAC animals developed HF. The left ventricle (LV) was severely dilated and the cardiac function as determined by fractional shortening and ejection fraction, were significantly decreased (Supplementary Fig. 6a–d). The SERCA2a protein levels were significantly decreased by ∼40% in TAC mice as compared with the sham-operated mice (Supplementary Fig. 6e). In addition, the positive chronotropic effects of dobutamine on myocardium were significantly abrogated in TAC mice (Supplementary Fig. 6f). Following cannulation of the carotid artery and external jugular vein, infusion of N106 or vehicle was performed as described in the Methods section. Pressure and volume measurements were made simultaneously and pressure volume curves were constructed as the vehicle or N106 were infused.

**Figure 4:**
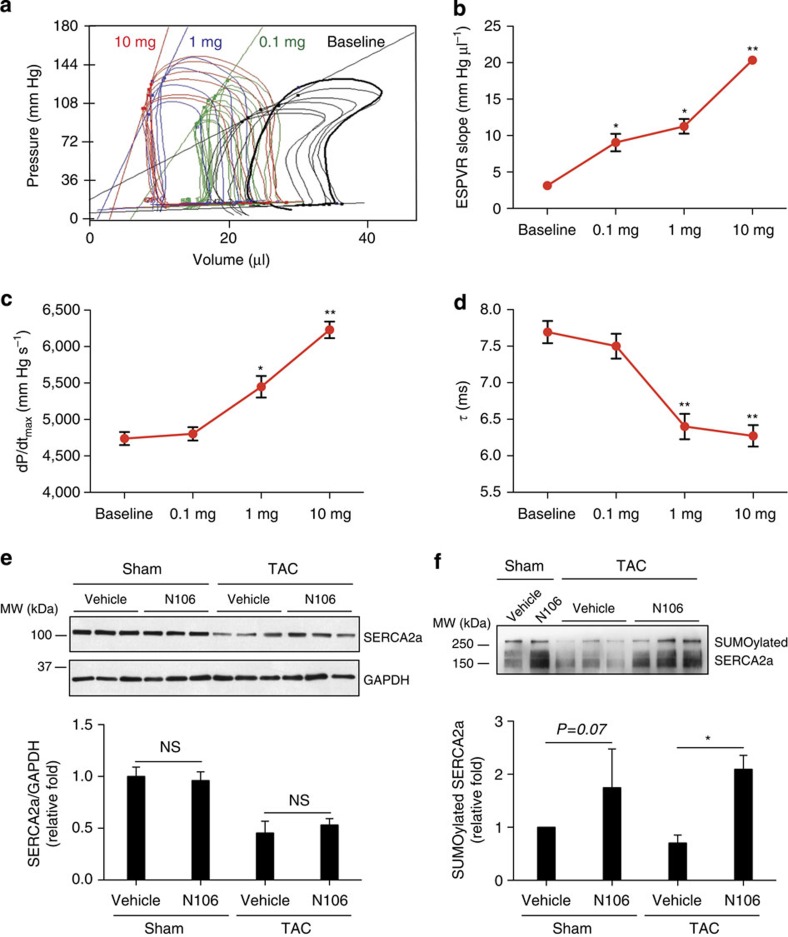
Infusion of N106 induces a sustained reversal of contractility in HF mouse. Beneficial effects of acute N106 treatment on cardiac function were shown in pressure overload-induced mouse model of HF. (**a**) Representative pressure volume loop showing that haemodynamic function of each increasing doses (0.1, 1.0 and 10 mg kg^−1^) of N106 compared with the baseline (with vehicle) at 30 min post treatment. Baseline in black; 0.1 mg kg^−1^ N106 compound in green; 1 mg kg^−1^ N106 compound in blue; 10 mg kg^−1^ N106 compound in red. (**b–d**) The ESPVR slope was steeper and maximal rate of contractility (dP/dt_max_) was increased when the heart received the N106 compound on dose-dependent manner. The relaxation parameter (*τ*) also enhanced in these conditions (*n*=10 independent mice per each N106 compound dose condition). (**e**) The effect of 10 mg kg^−1^ of N106 infusion on SERCA2a protein level was determined by western blot analysis. GAPDH (glyceraldehyde 3-phosphate) was used as a loading control. (**f**) SUMOylated SERCA2a level was examined by immunoprecipitation followed by western blot analysis. Data are represented as mean±s.e.m. *P≤0.05; ** *P*<0.001 (Student’s *t*-test) versus baseline. WB, western blot analysis; NS, not significant.

We found a beneficial effect on haemodynamic parameters following the infusion of N106. The end systolic pressure–volume relationship (ESPVR) in the LV was significantly steeper when N106 was infused compared with baseline (Fig. 4a). More importantly, infusion of N106 induced an increase in the index of contractility and ESPVR slope in a dose-dependent manner (Fig. 4b). Maximum dP/dt, an index of LV contractility, increased with increasing concentration of N106 (Fig. 4c), whereas *τ*, a parameter of relaxation, decreased, indicating enhanced relaxation (Fig. 4d). Changes in haemodynamic parameters are shown in Table 1. The vehicle infusion induced no change in the pressure–volume relationship and did not affect the index of contractility, ESPVR and +dP/dt_max_ in both sham and TAC mice (Supplementary Fig. 7). We showed enhanced contractility in normal cardiomyocytes after N106 treatment. However, in sham-operated groups there was an increasing trend; however, it was not statistically significant. Significant dose-dependent effects were not observed in those mice (Supplementary Fig. 8). This may be due to differences between the model systems (cell-based system versus *in-vivo* system) and bioavailability of the N106 compound *in vitro* versus *in vivo*.

Haemodynamic improvement occurred independent of the protein levels of SERCA2a and its regulatory protein. Western blot analyses revealed that SERCA2a protein levels were not significantly changed by N106 infusion (Fig. 4e). Neither protein levels nor phosphorylation states of phospholamban were changed by N106 treatment (Supplementary Fig. 9a,b). There were not significant changes in protein levels of cardiac ryanodine receptor and sodium–calcium exchanger ion channels in N106-treated mice (Supplementary Fig. 9c,d). However, in TAC hearts, SUMOylation of SERCA2a was significantly increased with the N106 treatment as compared with the vehicle control (Fig. 4f). There was an increasing trend in sham-operated group (*P*=0.07, Student’s *t*-test). SERCA2a’s ATPase activity was somewhat higher in N106-treated TAC hearts than vehicle-treated hearts (*P*=0.06, Student’s *t*-test; Supplementary Fig. 9e). These results indicate that N106 activates SUMOylation-dependent activation of SERCA2a proteins.

We further evaluated whether infusion of N106 would affect other SUMO-1 substrates. In TAC heart, N106 treatment significantly induced SUMOylated specificity protein 1 (Sp1) form. SUMOylation of Sp1 is known to decrease its protein stability^21^; however, protein levels of Sp1 were not changed following N106 treatment (Supplementary Fig. 10a). N106 had no significant effect on GATA4 SUMOylation and its protein levels (Supplementary Fig. 10b). The whole SUMO-1 conjugates were not significantly changed by N106 infusion (Supplementary Fig. 9f).

### Haemodynamic effects of N106 in the SERCA2a knockout mouse

In a second set of experiments, we tested whether N106 exerts its effects through SERCA2a. We used adult mice with an inducible cardiomyocyte-specific excision of the *Atp2a2* (*Serca2*) gene (Serca2 KO)^22^. Four weeks after induction of *Serca2* gene excision, the mice displayed a substantial reduction in systolic and diastolic function (Supplementary Fig. 11a). Ejection fraction was significantly lower in the Serca2 knockout (KO) mice compared with the WT control (Serca2 KO, 80.90±2.40%; WT, 95.4±0.11%, *P*<0.001, Student’s *t*-test; Supplementary Fig. 11b). In addition, the LV fractional shortening was significantly decreased (Serca2 KO, 43.80±0.79%; WT, 65.04±0.29%, *P*<0.001, Student’s *t*-test; Supplementary Fig. 11c) and the LV dimensions, as measured by LV end-diastolic internal diameters and LV end-systolic internal diameters, were significantly increased (*P*<0.001; Supplementary Fig. 11d,e). The decay constant, *τ*-value, was nearly 1.6-fold (Serca2 KO, 11.78±0.94 ms; WT, 7.0±0.25 ms, *P*<0.001, Student’s *t*-test) larger in the Serca2 KO mice compared with the WT control (Supplementary Fig. 11f). As expected, the expression of SERCA2a was completely abolished in the Serca2 KO mice (Supplementary Fig. 11g).

**Figure 5:**
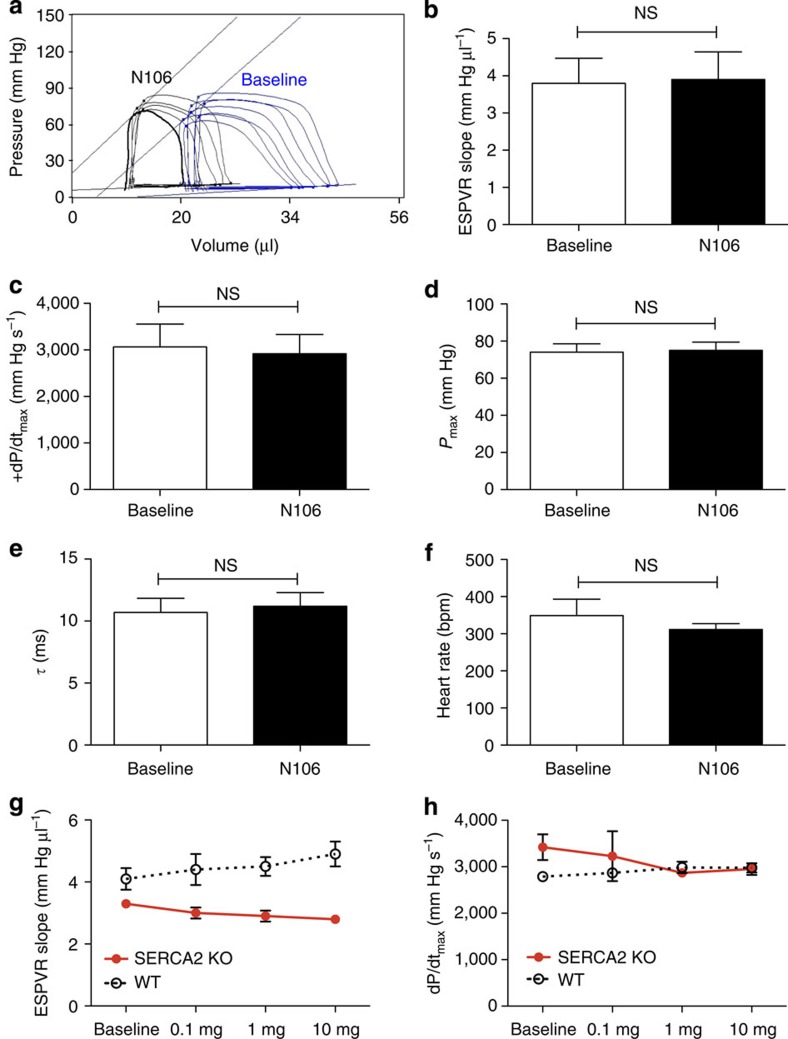
Lack of effects of N106 on haemodynamic parameters in cardiac-specific Serca2a knockout mice. Systolic and diastolic function was determined in conditional cardiac-specific Serca2a knockout mice by pressure–volume analysis. Four weeks after tamoxifen administration, the mice showed severe HF phenotype. There was no significant change in haemodynamic parameters with N106 treatment after a single dose (1 mg kg^−1^) (**a-f**) and different doses (0.1, 1 and 10 mg kg^−1^) (**g**,**h**). Data are represented as mean±s.e.m. *n*=3∼8 mice per each condition. NS, not significant; Student’s *t*-test.

As shown in Fig. 5 and Table 2, N106 did not induce any improvements in haemodynamic parameters in the Serca2 KO. ESPVR slope (*P*=0.17, versus baseline, Student’s *t*-test), +dP/dt_max_ (*P*=0.28, versus baseline, Student’s *t*-test), *P*_max_ (*P*=0.66 versus baseline, Student’s *t*-test), *τ* (*P*=0.10, versus baseline, Student’s *t*-test) and heart rate (HR; *P*=0.06, versus baseline, Student’s *t*-test) were not different in the presence of N106 (Fig. 5a–f). Furthermore, the dose-dependent enhancement of cardiac contractility by N106 disappeared in the Serca2 KO mice (Fig. 5g,h). Taken together, the animal data strongly suggest that N106 improves cardiac function through SERCA2a protein.

## Discussion

In this study, we demonstrated that small-molecule-mediated activation of SERCA2a SUMOylation provided direct beneficial effects on contractility in failing hearts. Based on its ability to SUMOylate SERCA2a, a specific compound (N106) was chosen. It improved contractility in a dose-dependent manner and calcium-transient property in isolated cardiomyocytes. In the pressure-overload-induced HF model, we found that N106 induced sustained reversal of contractility and was associated with an increased SERCA2a SUMOylation profile.

We tested the specificity of this novel compound N106 for SERCA2a, using molecular, cellular and animal model approaches. N106 seems to increase SUMOylation on SERCA2a with a subset of substrates, although it targets SUMO E1 enzyme. We showed that the extent of SUMOylation changes of individual proteins could be different in response to N106 treatment (Supplementary Fig. 10). One possibility to explain the distinct SUMOylation profiles between the substrates is a dependency on SUMO E3 ligase activity for their SUMOylation. RNF4 triggers Sp1 SUMOylation^23^, whereas PIAS1 serves as the SUMO E3 ligase for GATA4 (ref. 24). However, SUMOylation of SERCA2a does not require a SUMO E3 ligase. Another possibility is that N106-induced activation is not enough to achieve significant SUMOylation of broad ranges of substrate in this setting. In fact, the global SUMO-1 conjugates were not significantly increased in TAC hearts treated with N106 versus vehicle-treated hearts (Supplementary Fig. 9f). The strongest evidence that the beneficial effects of N106 are through SERCA2a comes from our results in the Serca2 KO mice, where N106 had no effects. However, SUMOylation of other myocardial targets aside from SERCA2a may also contribute to the reversal of cardiac dysfunction.

One concern about an agent that enhances SUMOylation is off-target effects including tumour growth. Several studies reported direct involvement of SUMO E1 in the setting of cancer. In patients with hepatocellular carcinoma, *UBA2* gene expression was higher and directly correlated with lower survival rate^25^. SUMO E1 was also reported to be an important factor for *myc*-dependent tumour growth in mice and there is a correlation between low *SUMO E1* gene expression and longer metastasis-free survival rate of patients with *myc*-high breast cancers^26^. Thus, we sought to determine whether N106 would have an effect on cancer cell growth. We performed the NCI-60 human tumour cell screen used by the National Cancer Institute’s Developmental Therapeutics Program (http://dtp.cancer.gov/docs/compare/compare.html). The NCI-60 set includes leukemia, lymphomas and carcinomas of ovarian, renal, breast, prostate, colon, lung and central nervous system origin. The results of the screen validated that the N106 did not induce any tumour cell growth and proliferation including *myc*-dependent human breast cancer cell line MDA-MB-231 (Supplementary Fig. 12). Even though the NCI-60 screen revealed that N106 does not increase tumour cell line growth, further *in-vivo* toxicology studies are warranted to diminish further perceived concerns for the proliferative potential for this pathway.

N106 exhibited a pharmacokinetic profile suitable for *in-vivo* evaluation in mice. In a murine model, the half-life of N106 was determined to be ∼65.4 min with a *C*_max_ of ∼2.24 μM when the mice received 10 mg kg^−1^ of N106 by intravenous injection. The oral bioavailability (F%) was 56% and 50%, and terminal elimination half-life (*t*_1/2_) was 19 min (Supplementary Fig. 13). This pharmacokinetic profile of N106 allows us to consider using N106 intravenously and orally as well. Further experiments will determine the most effective way of delivering this small molecule. Although the half-life of N106 *in vivo* is quite rapid, N106 increased cell contractility, calcium-transient SERCA2a’s ATPase activity and SUMOylation within 10 min of exposure, and these effects were sustained at 24 h in cardiomyocytes (Fig. 2). Thus, chronic studies need to be completed to evaluate long-term efficiency and toxicology of N106.

We also demonstrated the efficacy of N106 in a mouse model of HF. A limitation of using mice to study HF is related to significant differences in cardiac characteristics between mouse and human such as HR, small size, oxygen consumption and adrenergic receptor ratio^27^,^28^. Therefore, experiments in larger animal models and other models of HF will be essential to develop our discoveries from murine models into clinical therapies.

In summary, a number of experimental studies and now clinical trials have validated SERCA2a as an important target for the treatment of HF. In this study, we identified a small molecule that activates SERCA2a through SUMOylation and induces beneficial effects *in vitro* and *in vivo*. This first-in-class small-molecule activator may provide a novel therapeutic strategy for the treatment of HF.

## Methods

### Animals

All mice and rats were housed and treated in accordance with NIH and Institutional Animal Care and Use Committee guidelines and the used protocols were approved by the Icahn School of Medicine at Mount Sinai, Animal Care and Use Committees. Studies were conducted in male B6C3F1 mice aged 8–10 weeks (weight, 25∼30 g) purchased from Jackson Laboratories (Bar Harbor, ME). Male rats aged 3 months and weighting 180∼250 g were obtained from Jackson Laboratories.

### Cardiomyocyte isolation and cardiac physiology

Ventricular heart muscle cells were isolated from 180∼250 g male Sprague–Dawley rats as described^29^. The hearts were perfused sequentially with low-calcium buffer, enzyme solutions containing collagenases and proteases, and high-potassium solution. Next, the hearts were minced, filtered and resuspended in high-potassium solution. The cardiomyocytes were plated on laminin-coated plates and cultured in medium M199 (Life Technologies, CT, USA) containing proper supplements. The basic culture medium consisted of medium M199 with Earle’s salts, 5 mmol l^−1^ creatine (Sigma-Aldrich, Saint Louis, MO, USA), 2 mmol l^−1^ l-carnitine (Sigma-Aldrich), 5 mmol l^−**1**^ taurine (Sigma-Aldrich), 100 IU ml^−**1**^ penicillin (Life Technologies) and 100 μg ml^−**1**^ streptomycin (Life Technologies). At 4 h after plating, the culture medium was changed and small molecules were added. As indicators of cardiac functions, measurement of intracellular calcium and cell shortening were performed. In brief, the cardiomyocytes were loaded with the calcium indicator Fura-2 and stimulated at different frequencies (0.1∼3.0 Hz) using an external stimulator (Grass Technologies, Warwick, RI, USA), whereas a dual-excitation spectrofluorometer (IonOptix Inc., Milton, MA, USA) was used to record fluorescence emissions used to calculate intracellular calcium concentration. Cell shortening was measured using video microscopy motion-detector system.

### Transverse aortic constriction

B6C3F1 mice were acquired from Jackson Laboratories. Mice underwent TAC using a supraclavicular construction model as described^15^,^30^. TAC or sham surgery was performed in 8∼10-week-old male mice (25∼30 g). Mice were anaesthetized with intraperitoneal ketamine (95 mg kg^−1^) and xylaxine (5 mg kg^−1^), and ventilated with a tidal volume of 0.1 ml and a respiratory rate of 110 breaths per minute (Harvard Apparatus, MA, USA). A 2- to 3-mm longitudinal cut was made in the proximal portion of the sternum, allowing visualization of the aortic arch. The transverse aortic arch was ligated between the innominate and left common carotid arteries with an overlaying 27-gauge needle. The needle was immediately removed, leaving a discrete region of constriction. Animal sham group underwent a similar procedure without ligation. Only animals with fractional shortening < 50% were determined as being in HF and suitable for further study.

### Echocardiography

Mice were anaesthetized with intraperitoneal ketamine (100 μg g^−1^) for echocardiographic analysis. Transthoracic echocardiography was performed using a Vivid 7,000 equipped (GE Healthcare, Salt Lake City, UT, USA) with a H13L transducer (14 MHz). Two-dimensional and M-mode images were obtained in the short-axis view. The HR, LV end-diastolic internal diameter and LV end-systolic internal diameter were measured over the course of at least three repeated cardiac cycles. The fractional shortening and ejection fraction were then calculated.

### Invasive *P*–*V* loop measurement of cardiac function

One day after echocardiography, we performed *in-vivo* haemodynamics. The mice were anaesthetized with an intraperitoneal injection mixture of urethane (1 mg g^−1^), etomidate (10 μg g^−1^) and morphine (1 μg g^−1^), and were then intubated via a tracheotomy and mechanically ventilated at 7 μl g^−1^ tidal volume and 120 respirations per minute. A small incision was made in the apex and a French Millar cathether connected a high-fidelity pressure transducer (Scisense, London, ON, Canada) inserted into the LV. Haemodynamic recordings were performed after 5∼10 min of stable HR. The rate of pressure rise (+dP/dt) and maximal pressure, the time constant of isovolumic relaxation and HR were recorded and analysed. To determine the relative effects of the admittance and conductance techniques to contractility change induced by N106 compound infusion, whole haemodynamic function was recorded at both baseline and after administration of N106 compound through the external jugular vein. N106 compound was solubilized in 10% DMSO (Sigma-Aldrich), 10% Tween-80 (Sigma-Aldrich) and saline for *in-vivo* experiments. N106 or vehicle solutions were continuously infused through the external jugular vein at a rate of 50 μl min^−1^ for 2 min in each dose. Whole haemodynamic functions of increasing dose of N106 of each mouse were recorded for ∼4 h. Each dose of N106 solution was infused after back to baseline of heart function.

### Western blot analysis

Protein extracts (20∼50 μg) were obtained from isolated cardiomyocytes, which was lysed in RIPA buffer (R&D Systems Inc., MN, USA) contained protease inhibitors (Complete mini, Roche Diagnostics, IN, USA) and 20 mM NEM (Sigma-Aldrich). For SERCA2a SUMOylation assays, cardiac protein extracts were immunoprecipitated with a SUMO-1 agarose resin (Santa Cruz Biotechnology Inc., CA, USA) for overnight at 4 °C. The resins were washed with cold lysis buffer three times. The immunocomplexes were then resolved by SDS–PAGE and western blot analysis was performed using a SERCA2a-specific antibody. For general western blotting, cardiac proteins (10∼50 μg) were separated on SDS–PAGE gel, transferred to nitrocellulose membrane (Bio-Rad, CA, USA) and blotted with the following primary antibodies: SUMO-1 (1:1,000, R&D Systems Inc.), Ubc9 (1:1,000, R&D Systems Inc.), SAE2 (1:1,000, Abcam Inc., MA, USA), SERCA2a (1:3,000, 21st Century Biochemicals, MA, USA), PLN (1:5,000, Badrilla Ltd., UK), PLN phosphorylation at serine-16 (1:5,000, Badrilla Ltd.), RyR2 (1:5,000, Abcam Inc.), NCX (1:1,000, Novus Biologicals, CO, USA), glyceraldehyde 3-phosphate (1:10,000, Sigma-Aldrich), GATA4 (1:1,000, Millipore, NY, USA) and Sp1 (1:500, Millipore). Secondary antibodies anti-mouse IgG-HRP (1:10,000) and anti-rabbit IgG-HRP (1:10,000) were purchased from Sigma-Aldrich. The immunoreactive bands were visualized by a supersignal chemiluminescence detection reagent (Pierce Biotechnology, IL, USA). Full scans of western blots are presented in Supplementary Fig. 14.

### Recombinant protein expression and purification

Human SAE1 and SAE2 were amplified by PCR from complementary DNA obtained from OriGene Tech (San Francisco, CA, USA) and cloned into the GST expression vector, pGEX-6P-3 (GE Healthcare Life Sciences, Piscataway, NJ). Expression vectors coding for WT SAE1/SAE2 were constructed as described^31^. pGEX-6P-SAE2 plasmid for expression of SAE2 was cloned and constructed by PCR amplification of SAE2 cDNA with the primers 5′-CGGGATCCATGGCACTGTCGCGGGGGCTG-3′ and 5′-CGGAATTCTCAATCTAATGCTATGACATC-3′. The PCR product was cleaved with BamHI and EcoRI before insertion into similarly cleaved pGEX-6P. A plasmid for translationally linked expression of GST-SAE2/SAE1 was constructed by PCR amplification of SAE1 cDNA with primers containing a ribosome-binding site and EcoRI or SalI restriction sites (5′-CGGAATCCATTAAAAGGAGAAATTAACT ATGGTGGAGAAGGAGGAG-3′ and 5′-ACGCGTCGACTCACTTGGGGCCAAGGCACTC-3′). The PCR product was cloned as EcoRI and SalI insert in pGEX-6P-SAE2, to form the GST-SAE2/SAE1 expression plasmid. Mutant SUMO E1s were generated by PCR-based mutagenesis (QuikChange mutagenesis kit, Stratagene, CA, USA) as described by the manufacturer, using primers designed for each mutation (Q312A, 5′-GGTTGGAGGGATTTTGGCAGCGGAAATTGTGAAGGCCCTG-3′, 5′-CTGAGACAGGGCCTTCACAATTTCCGCTGCCAAAATCCCTC-3′; V315F, 5′-TTGGCACAGGAAATTTTTAAGGCCCTGTCTCAG-3′, 5′-TGAGACAGGGCCTTAAAAATTTCCTGTGCCAAA-3′). All substitution were confirmed by DNA sequencing. Recombinant protein SUMO E1s were produced in *Escherichia coli* BL21 from a bicistronic constructs of GST-SAE2 and untagged SAE1. GST-fusion SAE2/SAE1 proteins were purified by affinity chromatography on glutathione-Sepharose beads as outlined by the manufacturer (Amersham Pharmacia Biotech, Uppsala, Sweden).

### SUMO E1 activity assays

Biochemical activity of SUMO-specific E1 enzymes was examined by ATPase assay and *in-vitro* gel-shifting assays. ATP hydrolysis activity of E1 enzymes was measured by using pyrophosphate assay kit (Abcam Inc.). E1 (0.05 μM), 0.25 μM E2, 12.5 μM SUMO-1 and 1 mM ATP with N106 or DMSO were used for the assays. The E1-thioester formation was analysed in reaction mixtures containing 0.05 μM E1, 12.5 μM SUMO-1 and 1 mM ATP with N106 or DMSO. The E1-dependent E2-SUMO-1 thioester formation was assessed using reaction containing 0.05 μM E1, 0.25 μM E2, 12.5 μM SUMO-1 and 1 mM ATP with N106 or DMSO. Assays were incubated at 37 °C for 30 min and were mixed with non-reducing sample buffer, and analysed in non-reducing condition. Western blotting using antibodies against SAE2 and Ubc9 detected the SUMO conjugations. To determine the effect of N106 on ubiquitin-activating enzyme E1 activity, similar assays were performed (Supplementary Fig.6). Inorganic pyrophosphate detection assays, ubiquitin-E1 conjugation and ubiquitin-E2 conjugation were performed as previously described^32^. Human recombinant proteins (WT SAE1/SAE2, Ubc9, SUMO-1, Ubiquitin and UbcH5A) and reaction buffers were purchased from R&D Systems. N106 stock solution for *in-vitro* assays was prepared in DMSO (Sigma-Aldrich).

### SERCA2a ATPase activity assay

SERCA2a ATPase activity was determined using a coupled enzymes assay including pyruvate kinase/lactate dehydrogenase/NADH-coupled reactions. Microsomes were enriched by differential density centrifugation. The cardiac cells and tissues were homogenized on ice in homogenization buffer (250 mM sucrose (Sigma-Aldrich), 5 mM HEPES pH7.0 (Sigma-Aldrich), 1 mM phenylmethyl sulfonyl fluoride (Sigma-Aldrich), protease inhibitor cocktail (Roche Life Science, IN, USA)). The homogenates were centrifuged at 1,000*g* for 10 min at 4 °C, then the supernatants were collected and centrifuged at 12,000*g* for 15 min at 4 °C. The supernatants were transferred to ultracentrifuge tubes and centrifuged for 1 h at 140,000*g* at 4 °C (Optima XE-100, Beckman Coulter). The pellets were resuspended in homogenization buffer to determine protein concentration and calcium-dependent ATPase activity assay. Protein concentration was measured with a 2-D Quant Kit (GE Healthcare Life Sciences, PA, USA). The ATPase activity were measured at 25 °C in 50 mM MOPS (Boston Bioproducts, MA, USA), 100 mM KCl (Boston Bioproducts), 5 mM MgCl_2_ (Boston Bioproducts), 1 mM EDTA (Boston Bioproducts) at pCa 5.0 by using a coupled enzyme system consisting of pyruvate kinase and lactate dehydrogenase, and monitoring the oxidation of the reduced form of NADH at 340 nM.

### Single-dose pharmacokinetic profile of N106

The male mice were received with a small molecule N106 at 10 mg kg^−1^ intravenously and 10 mg kg^−1^ orally (PO). The bloods were corrected at 30 min, 1, 2 and 4 h after administration intravenously and at 30 min, 1, 2 and 4 h after treatment for oral administration studies. Plasma samples from these studies were extracted by plasma separator tube containing lithium heparin (BD Microcontainer, NJ, USA). Plasma concentrations of N106 were determined by HPLC analysis and pharmacokinetic parameters. Three mice for each time point were used.

### Statistical analysis

Data represents mean value±s.e.m. *P*-values calculated using Student’s *t*-test for the change from baseline before treating with compound or vehicle control. Statistical analysis was performed using GraphPad Prism software (GraphPad Software, Inc., CA, USA).

## 

## Additional information

**How to cite this article:** Kho, C. *et al*. Small-molecule activation of SERCA2a SUMOylation for the treatment of heart failure. *Nat. Commun.* 6:7229 doi: 10.1038/ncomms8229 (2015).

## Supplementary Material

Supplementary Information Supplementary Figures 1-14

## Figures and Tables

**Table 1:** Haemodynamic parameters in N106-infused TAC mice.

	Baseline	0.1 mg kg^−1^	1 mg kg^−1^	10 mg kg^−1^
ESPVR slope (mm Hg μl^−1^)	3.13±0.09	9.04±1.20*	11.26±1.00*	20.3±0.41**
+dP/dt_max_ (mm Hg s^−1^)	4738.76±68.90	4803.33±91.78	5448.83±147.09*	6229.41±103.23**
*P*_max_ (mm Hg)	111.09±1.86	115.11±1.27	123.66±5.28*	124.50±4.26*
*τ* (ms)	7.69±0.13	7.50±0.15	6.40±0.15*	6.27±0.13**
HR (bpm)	422.13±10.23	417.11±4.54	422.33±3.79	455.08±5.62

+dP/dt_max_ LV maximal contraction rate (inotropic response); ESPVR, end systolic pressure–volume relationship; HR, heart rate; LV, left ventricle; N106, *N*-(4-methoxybenzo[d]thiazol-2-yl)-5-(4-methoxyphenyl)-1,3,4-oxadiazol-2-amine; *P*_max_, maximal pressure; TAC, transverse aortic constriction; *τ*, time constant of isovolumetric relaxation

N106 treatment improves LV systolic function in a dose-dependent manner. Data are represented as mean±s.e.m. *P≤0.05; **P<0.001 versus baseline (*n*=10 mice per each group, Student’s *t*-test).

**Table 2:** Haemodynamic parameters in N106-infused *Serca2a* knockout mice.

	Baseline (*n*=3)	Vehicle (*n*=3)	Baseline (*n*=8)	N106 (*n*=8)
ESPVR slope (mm Hg μl^−1^)	3.6±0.8	3.5±0.5	3.8±0.6	3.9±0.7
+dP/dt_max_ (mm Hg s^−1^)	2360.7±597.0	2023.6±674.9	3064.2±489.5	2917.8±412.9
*P*_max_ (mm Hg)	62.9±6.3	67.4±11.8	74.0±4.5	75.0±4.4
*τ* (ms)	10.1±2.0	10.2±1.1	10.7±1.1	11.2±1.0
HR (bpm)	315.2±34.6	291.5±36.3	348.8±44.1	311.2±16.1

+dP/dt_max_ LV maximal contraction rate (inotropic response); ESPVR, end systolic pressure–volume relationship; HR, heart rate; N106, *N*-(4-methoxybenzo[d]thiazol-2-yl)-5-(4-methoxyphenyl)-1,3,4-oxadiazol-2-amine; *P*_max_, maximal pressure; TAC, transverse aortic constriction; *τ*, time constant of isovolumetric relaxation

Eight-week-old male cardiac-specific Serca2a-deficient mice received tamoxifen intraperitoneally (1 mg per day for 4 days). Four weeks after, ESPVR slope, maximal pressure, +dP/dt_max_, *τ* and HR were recorded both at baseline and in response to 1 mg kg^−1^ of N106 administered via cannulation of the right internal jugular vein. Either vehicle or N106 had no effects on haemodynamic parameters in these mice. Data are represented as mean±s.e.m. Statistics were performed using a Student’s *t*-test.
