# Supplementary material for: Small-molecule activation of SERCA2a SUMOylation for the treatment of heart failure
Source: Nat Commun. 2015 Jun 12;6:7229. doi: 10.1038/ncomms8229 (PMC4467461; doi:10.1038/ncomms8229)
Supplement: Supplementary Information — Supplementary Figures 1-14 [file ncomms8229-s1.pdf]

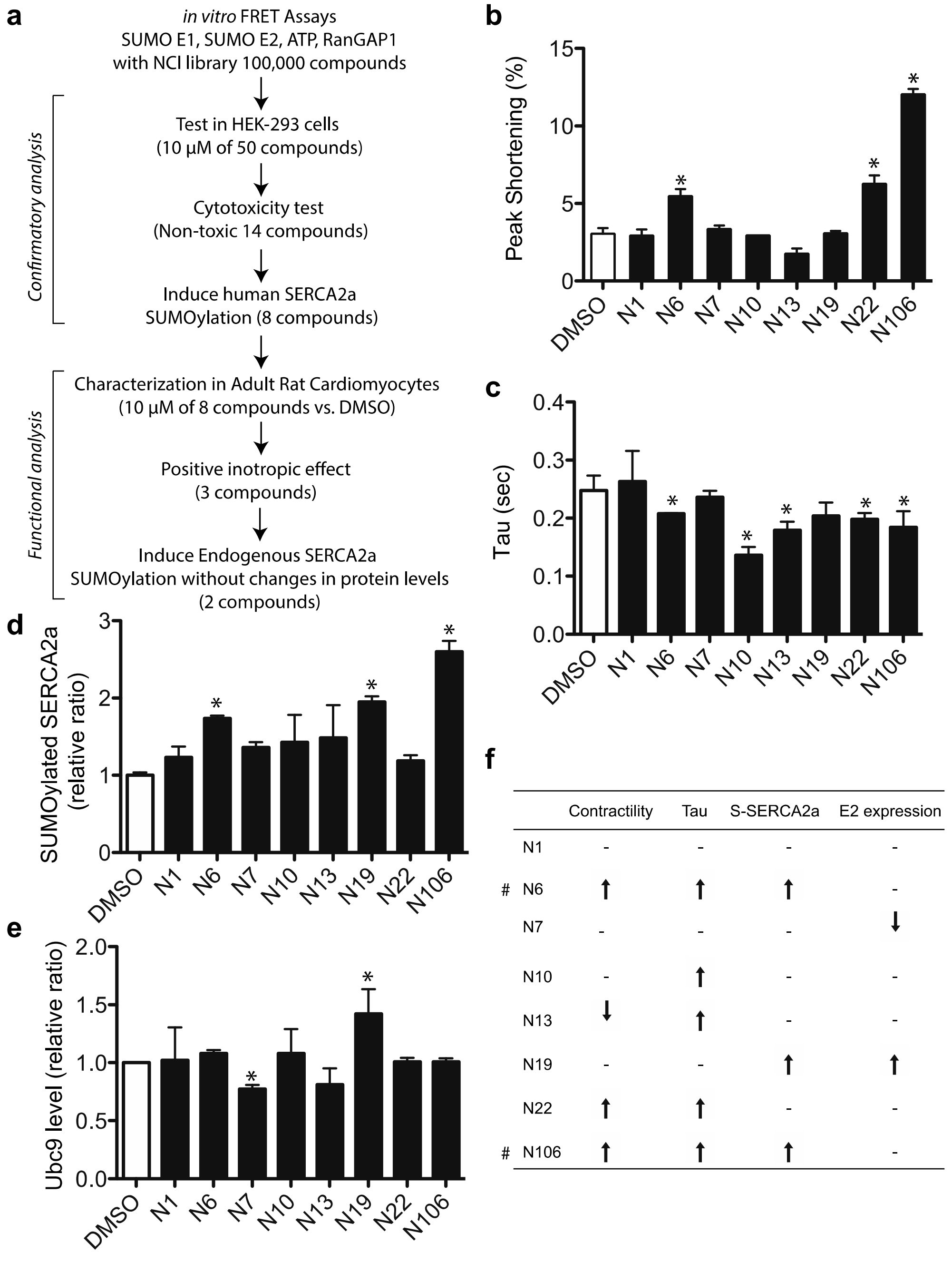

Supplementary Figure 1. Identification of potent SUMOylation activators. (**a**) Workflow describing the screening strategy to identify small molecules that increase SERCA2a SUMOylation and SERCA2a function. To confirm the initial FRET assay, HEK-293 cells were co-transfected with SERCA2a and YFP-SUMO-1 for 24 hours. The transfected HEK-293 cells were incubated with 10 µM of the candidate compounds for 24 hours. Cytotoxicity was evaluated by trypan blue assay. The level of SERCA2a SUMOylation was measured by immunoprecipitation and Western blot analysis. Eight compounds were selected for functional analysis. Adult ventricular cardiomyocytes were isolated from Sprague-Dawley rats and treated

with the small molecules at 10 μM for 24 hours. The mechanical properties of cadiomyocytes treated with small molecules were determined: peak shortening (**b**) and calcium transient (**c**). Molecular analysis was performed to determine the effects of the small molecules on levels of endogenous SERCA2a SUMOylation (**d**) and protein levels of E2 conjugation enzyme, Ubc9 (**e**). Panel (**f**) depicts the screening strategy. In this study, we focused on the small molecules that activate SERCA2a SUMOylation and that have positive effects on cardiomyocyte function. Compounds N6 and N106 were identified as lead compounds as activators of SERCA2a SUMOylation and enhancers of cardiomyocyte contractility. DMSO was used as vehicle control. # indicates the potential molecules. Data are represented as mean ± SEM. * $p \leq 0.05$ (Student's *t*-test). The conformational screen was performed once (**a**); subsequent analyses were performed three (**b,c**) ($n = 25$ cells per heart, three different hearts) or two times (**d,e**).

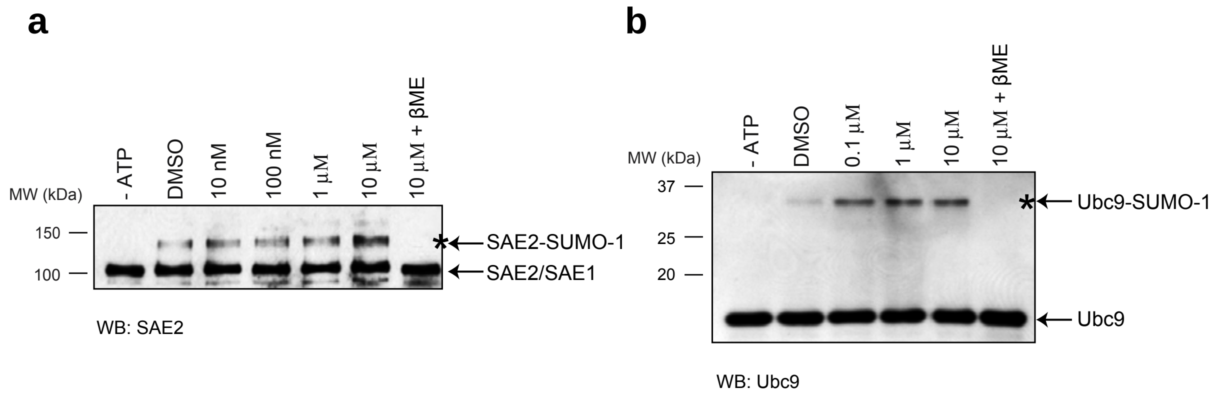

Supplementary Figure 2. *in vitro* β-mercaptoethanol-conjugation sensitive assays (a) Recombinant SUMO E1 (0.05 μM), SUMO-1 (12.5 μM), and ATP (1 mM) were co-incubated with increasing concentration of N106 for 30 minutes at 37°C. The reaction mixtures were separated on 7.5% SDS-PAGE gel under non-reducing or reducing conditions. The E1-SUMO-1 conjugation was detected followed by Western blotting with anti-SAE2 antibody. (**b**) Recombinant E1 (0.05 μM), recombinant E2 Ubc9 (0.25 μM), SUMO-1 (12.5 μM), and ATP (1 mM) was incubated without or with increasing concentration of N106 for 30 minutes at 37°C. The E2-SUM-1 conjugation was detected by Western blotting with anti-Ubc9 antibody. In the presence of 5 mM β-mercaptoethanol (βME), the SUMO-1 conjugations (*) were blocked indicating a thioester bond.

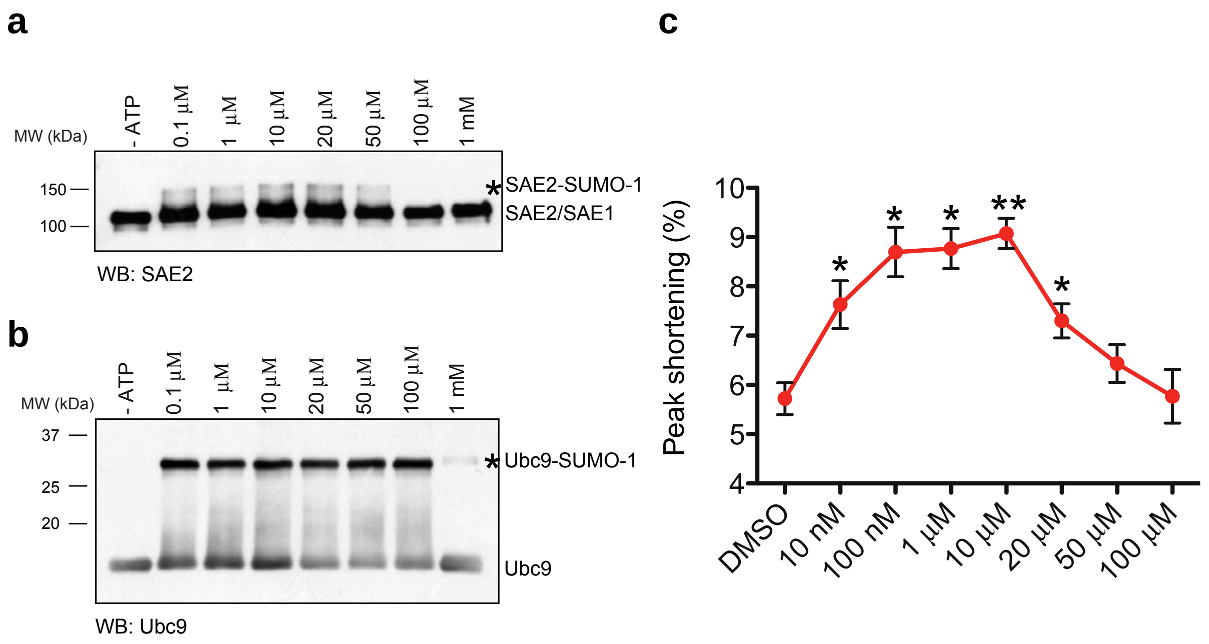

Supplementary Figure 3. *in vitro* measurement of maximum tolerated doses of N106. (a) The E1-SUMO-1 conjugation was induced with recombinant SUMO E1 (0.05 µM), SUMO-1 (12.5 µM), and ATP (1 mM) with increasing concentration of N106 for 30 minutes at 37°C. E2-SUMO-1 conjugation was performed with recombinant E1 (0.05 µM), recombinant E2 Ubc9 (0.25 µM), SUMO-1 (12.5 µM), and ATP (1 mM) with increasing concentration of N106 for 30 minutes at 37°C. The reaction mixtures were separated on SDS-PAGE gel under non-reducing conditions. The E1-SUMO-1 conjugation (**a,** *) and E1 dependent E2 -SUMO-1 conjugation (**b**, *) was detected by Western blotting with indicated antibodies. (**b**) The effect of N106 compound on contractility was determined by using the same set of cardiomyocytes. Data are represented as mean ± SEM. * $p \leq 0.05$ ($n = 25$ cardiomyocytes per group, three different hearts); ** $p < 0.001$ from DMSO control (Student's *t*-test).

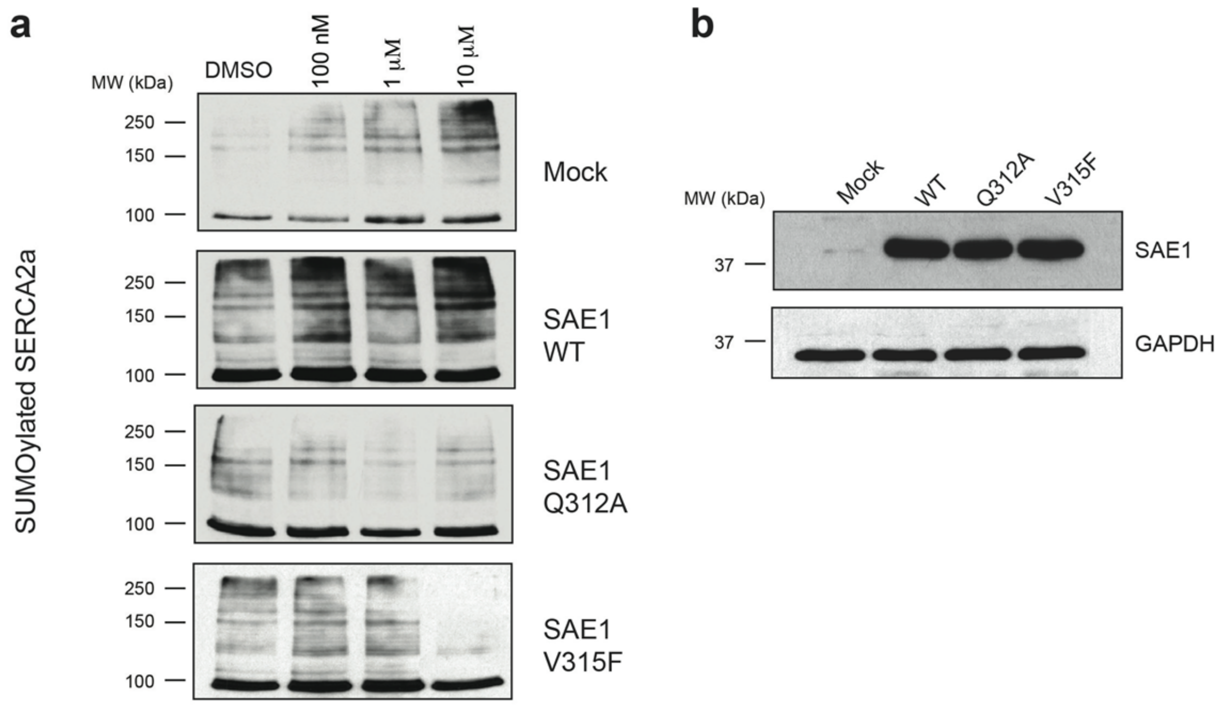

Supplementary Figure 4. Effects of overexpressing SAE1 mutants on N106-induced SERCA2a SUMOylation. (**a**) HEK-293 cells were co-transfected with SERCA2a and SAE1 wild type or SAE1 mutants (Q312A and V315F) for 24 hours. And then the transfecting HEK-293 cells were incubated with different concentration of N106 for 24 hours. The level of SERCA2a SUMOylation was determined by immunoprecipitation and following with Western blotting. **(b**) The levels of SAE1 overexpression were determined by Western blotting with anti-SAE1 antibody.

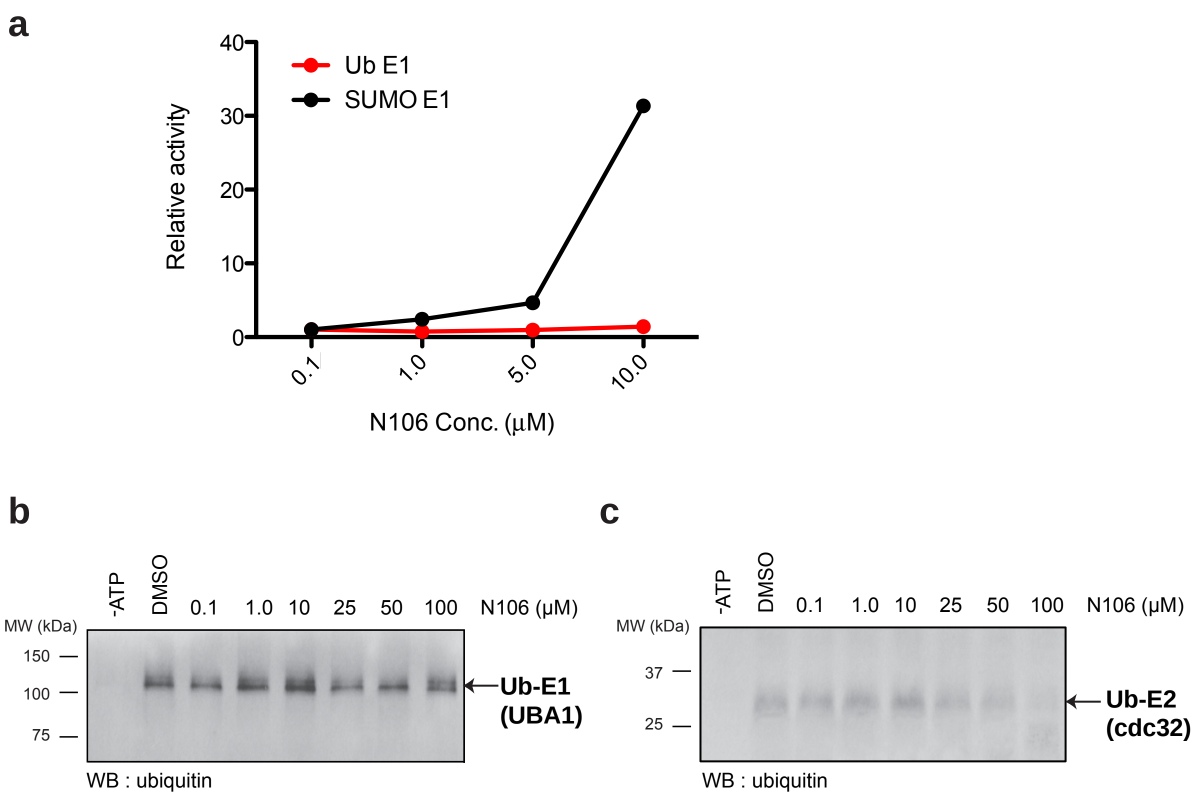

Supplementary Figure 5. No effect of N106 on ubiquitin activating E1 enzyme. Ubiquitin activating E1 enzyme activity was determined by ATP hydrolysis and *in vitro* conjugation assays. **(a)** Recombinant human ubiquitin specific E1 was incubated with the human cdc32 E2, human ubiquitin, and ATP in the presence of increasing concentrations of N106 for 30 minutes at 37°C. Inorganic pyrophosphate resulting from ATP hydrolysis in E1-catalazed ubiquitin activity was quantified with the use of a fluorogenic pyrophosphate assay kit. **(b)** Ubiquitin specific E1 enzyme and ubiquitin were incubated with increasing concentration of N106 for 30 minutes at 37°C. Thioester bond formation between ubiquitin specific E1 enzyme and ubiquitin were detected by Western blot analysis. **(c)** Ubiquitin specific E1, E2, ubiquitin, and ATP were incubated with or without increasing concentration of N106 for 30 minutes at 37°C. E1-dependent thioester bond formation between E2 and ubiquitin was detected by Western blotting with anti-ubiquitin antibody. Two-independent experiments were performed.

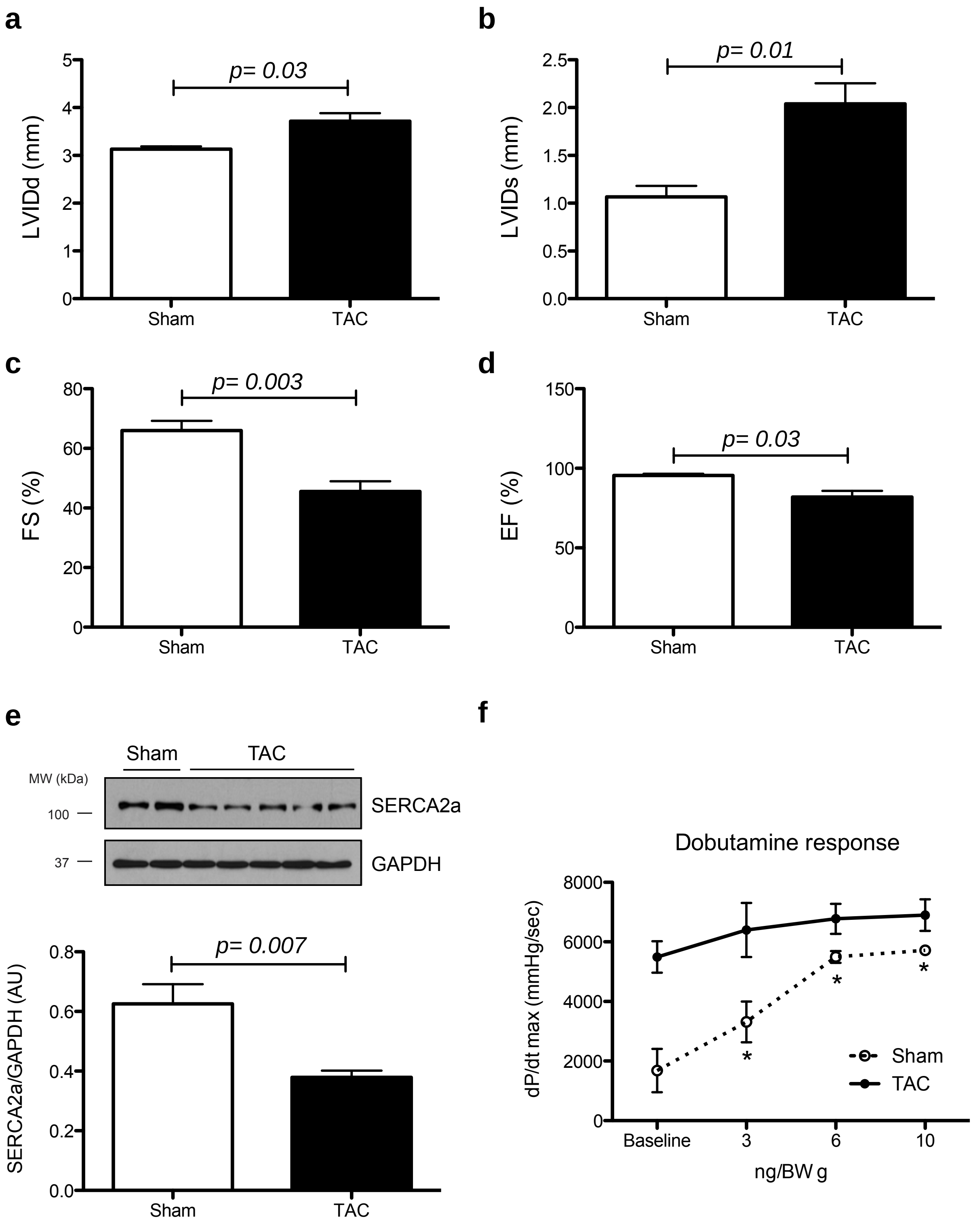

Supplementary Figure 6. TAC-induced heart failure animal characterization. Two months after TAC operation, mice developed heart failure. **(a-d)** Echocardiographic parameters of heart failure animals. LV chamber dimensions and LV systolic function were calculated at 2 months of

TAC mice and sham control. $n = 5$ (TAC) and $n = 3$ (sham operated). LVIDd, LV internal diameter during diastole; LVIDs, LV internal diameter during systole; FS, fractional shortening; EF, ejection fraction. **(e)** SERCA2a protein levels were detected by Western blot analysis. 15 μg of tissue extracts was separated on 7.5% SDS-PAGE gels, transferred to nitrocellulose membrane and blotted with anti-SERCA2a antibody. Band intensity was quantified by Image *J* software. The amount of GAPDH was used as a normalization control. **(f)** Dobutamine was infused to test contractile response to β-agonist stimulation in TAC and sham operated animals. LV pressure rise rate (+dP/dt max) was measured in response to different dose of dobutamine stimulation. Dose-dependent dobutamine response was blunted in TAC animals. $n = 3$ mice per each dose. Data are represented as mean ± SEM. * $p < 0.05$ versus baseline (Student's *t*-test).

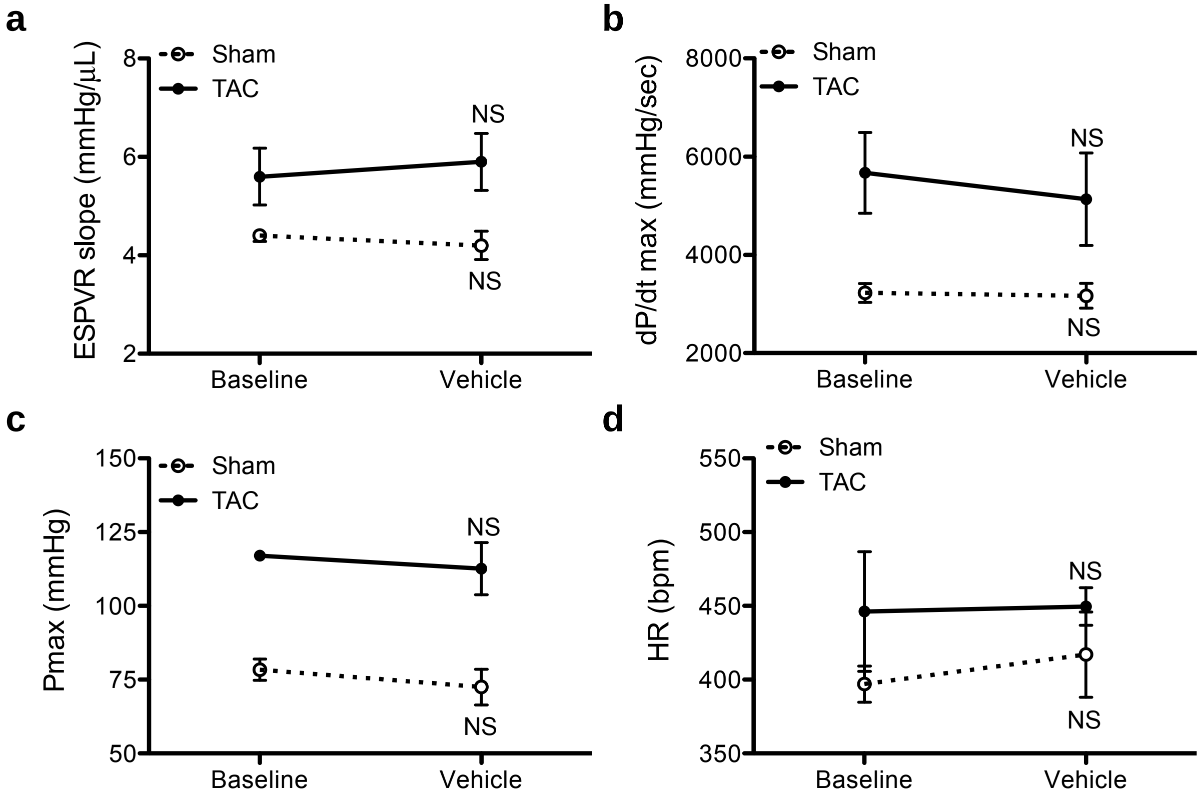

Supplementary Figure 7. No effect of vehicle infusion on hemodynamic parameters in mouse model. No effect of vehicle treatment on hemodynamic parameters in both TAC mice (2 months post operation) and age-matched sham-operated mice. ESPVR slope (**a**), dP/dt max (**b**), maximal pressure (**c**), and heart rate (**d**) were not significantly altered in both TAC (solid line, n = 6) and sham-operated mice (n = 3) after receiving vehicle solution compared to the baseline. Data are represented as mean ± SEM. Student's t-test (baseline versus vehicle treated mice). NS, not significant.

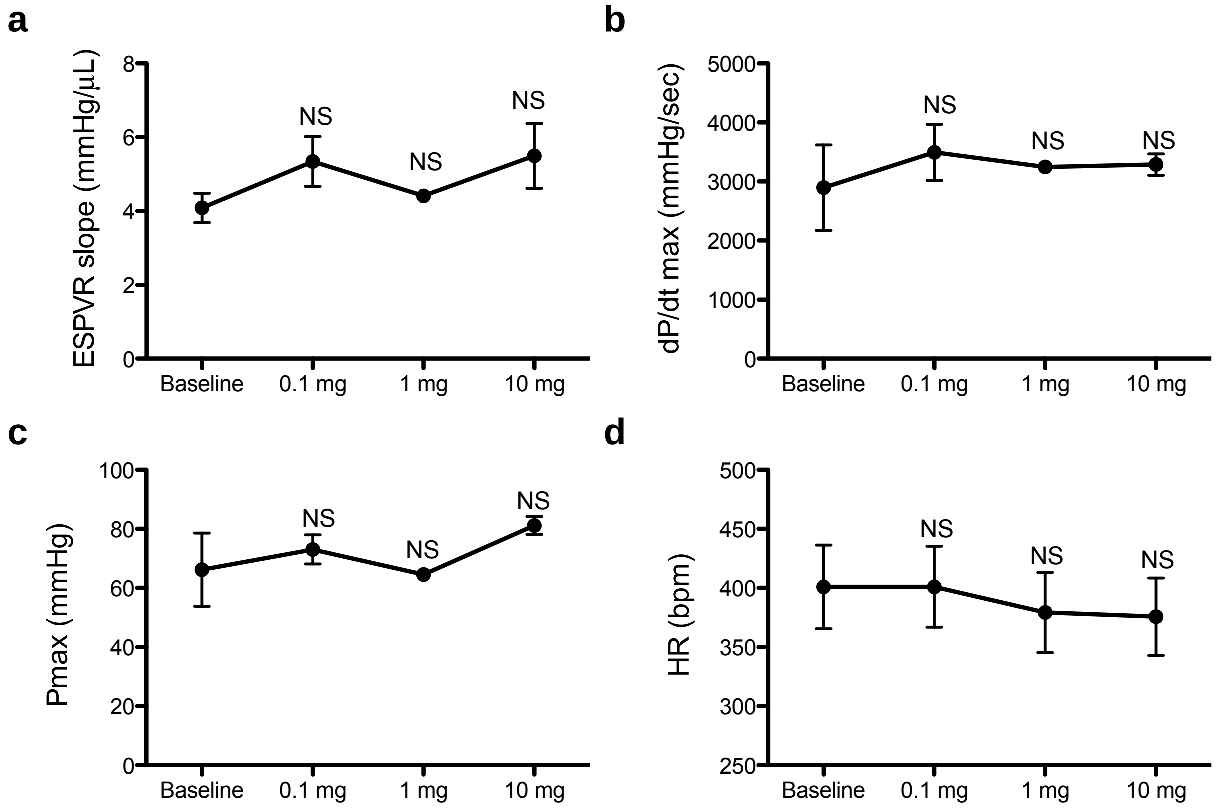

Supplementary Figure 8. Hemodynamic measurements in sham-operated mice treated with increasing doses of N106. In sham mice, N106 infusion had no significant effect on ESPVR slope (**e**), dP/dt max (**f**), maximal pressure (**g**) and heart rate (**h**). Data are represented as mean ± SEM. *n* = 3 mice for each dose. Student's *t*-test. NS, not significant.

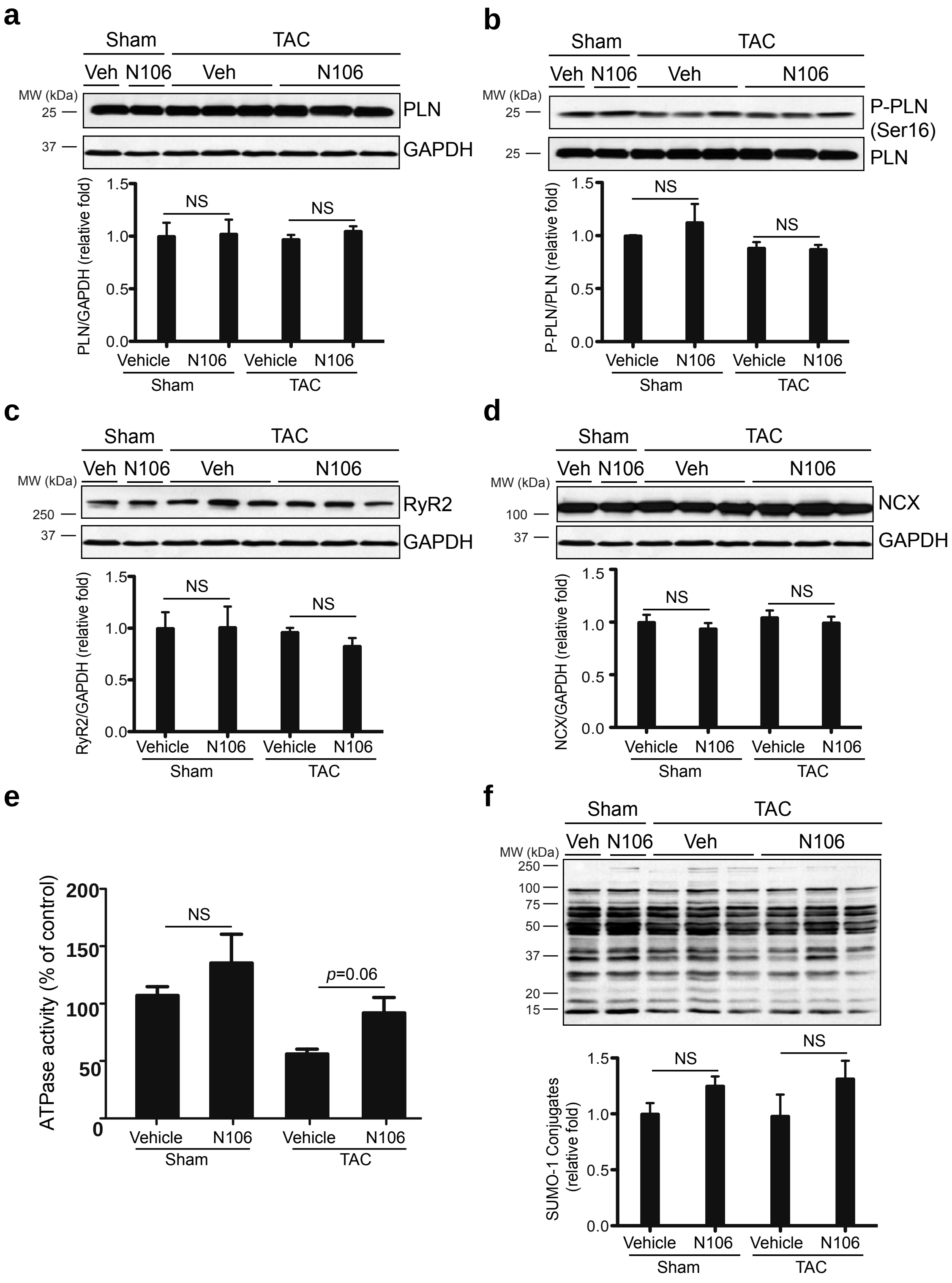

Supplementary Figure 9. Molecular analysis in N106 treated mice. (a-d) Representative western blots and protein quantification of select cardiac proteins. Mice heart tissue samples were obtained after external jugular vein infusion with vehicle or 10 mg·kg^{-1}of N106 from sham and TAC mice. The experiments shown are representative of three independent Western blots with

similar results. **(e)** The enzyme activity of SERCA2a was determined by using the same set of heart tissue samples. **(f)** SUMO-1 conjugate was measured by Western blot analysis with an anti-SUMO-1 antibody. Band intensity was quantified by Image *J* software. The amount of GAPDH was used as a normalization control. Data are represented as mean ± SEM. Student's *t*-test. NS, not significant. PLN, phospholamban; P-PLN, phosphorylated phospholamban at serine 16 residue; RyR2, ryanodine receptor 2; NCX, sodium-calcium exchanger; Veh, vehicle treated group (n = 3 mice); N106, N106 treated group (n = 3 mice).

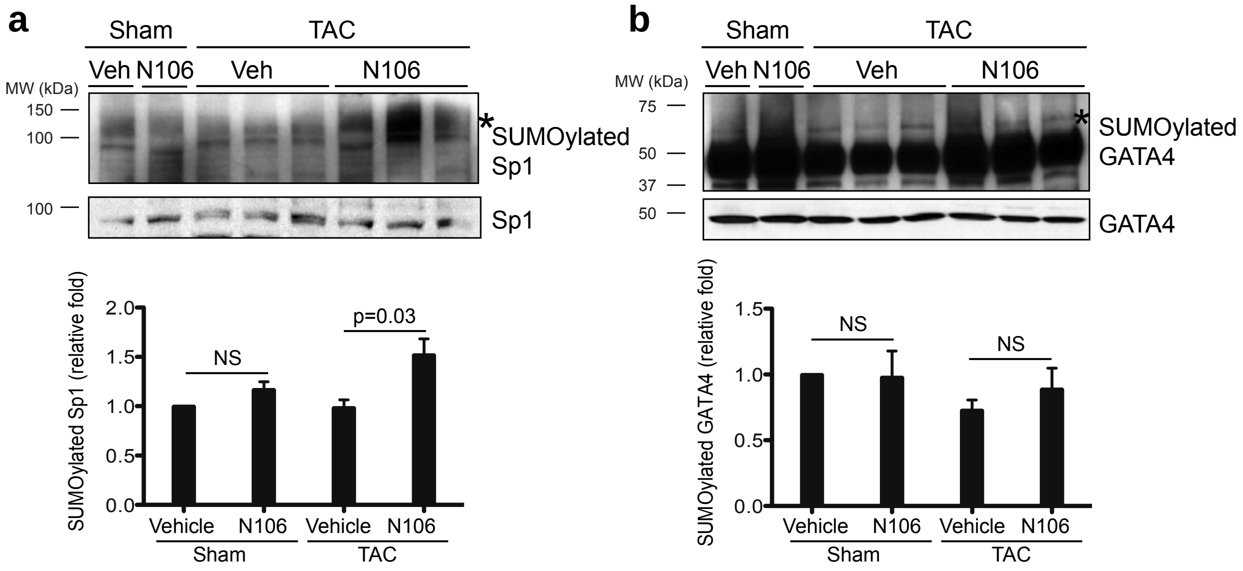

Supplementary Figure 10. Effect of N106 infusion on SUMOylation of two known substrates. SUMOylation of Sp1 (**a**) and GATA4 (**b**) were analyzed in cardiac tissues treated with 10 mg·kg^{-1} of N106 or vehicle. Band intensity was quantified by Image *J* software. Data are represented as mean ± SEM. Student's *t*-test. NS, not significant. Veh, vehicle treated group (*n* = 3 mice); N106, N106 treated group (*n* = 3 mice). * indicates SUMOylated forms.

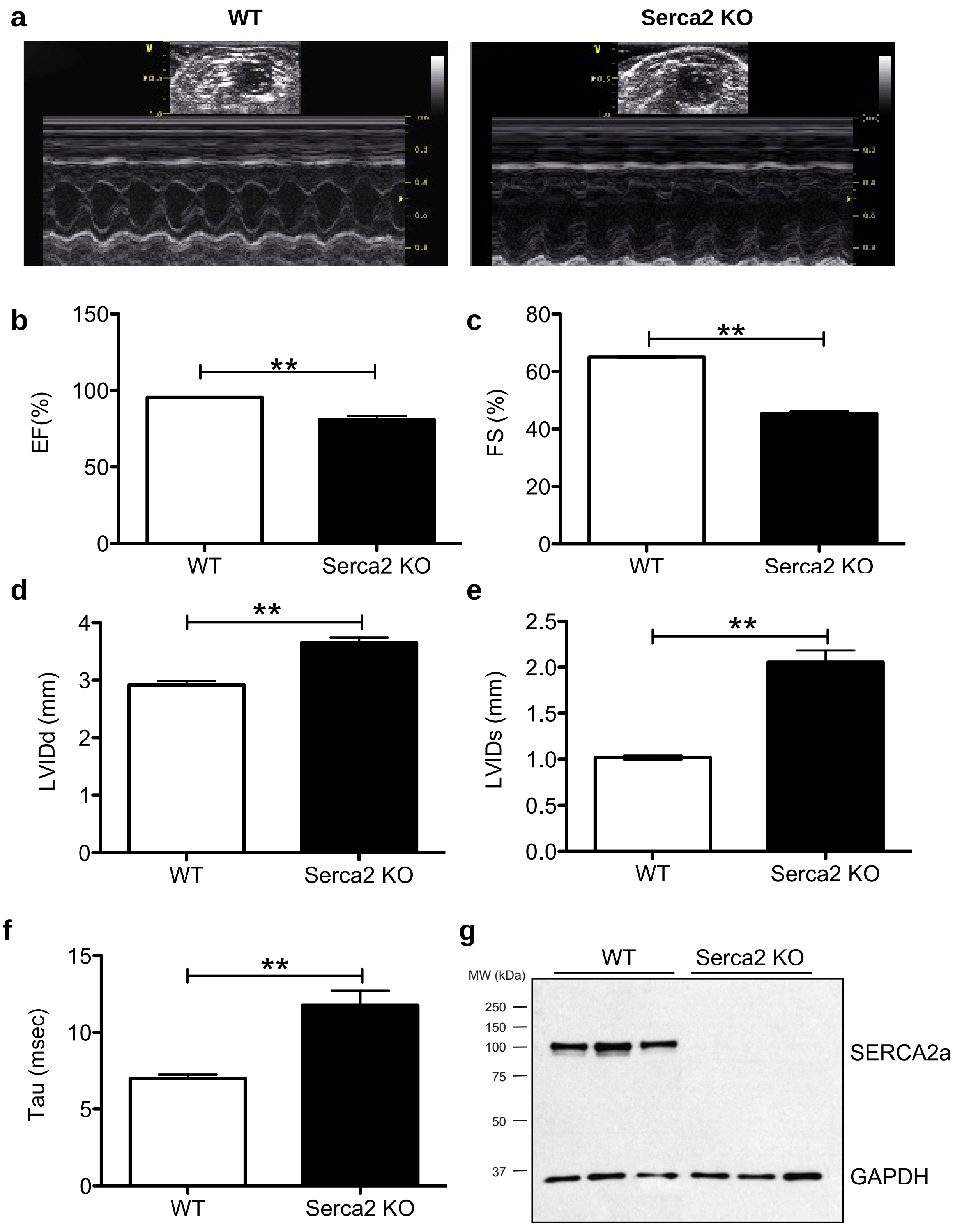

Supplementary Figure 11. Characterization of Serca2a knockout mice. Conditional cardiomyocyte-specific *Serca*2 knockout mouse model has been previously described[20]. 8 weeks old male mice were injected intraperitoneally with tamoxifen to induce *Serca*2 gene disruption.

Four weeks after tamoxifen administration, the Serca2 knockout mice developed heart failure. **(a-e)** Echocardiographic M-mode images showing LV dilation in Serca2 knockout mice (top). Ejection fraction (EF) and fractional shortening (FS), expressed as percentages determined the cardiac function. LVIDd and LVIDs determined the LV dimension in Serca2 knockout mice and wild type control mice ($n = 5$). **(f)** Tau values, an indicator of SERCA2a activity, were obtained from hemodynamic analysis ($n = 5$). LV relaxation constant was slow following Serca2 knockout. **(g)** Western blot analysis of SERCA2a protein levels in tamoxifen induced Serca2 knockout mice and wild type control. 15 μg of cardiac homogenates obtained from wild type and Serca2 knockout mice were separated on 7.5% SDS-PAGE gel and blotted with anti-SERCA2a specific antibody. GAPDH was used as a loading control. Data are represented as mean ± SEM. ** $p < 0.001$ versus wild-type control (Student's *t*-test).

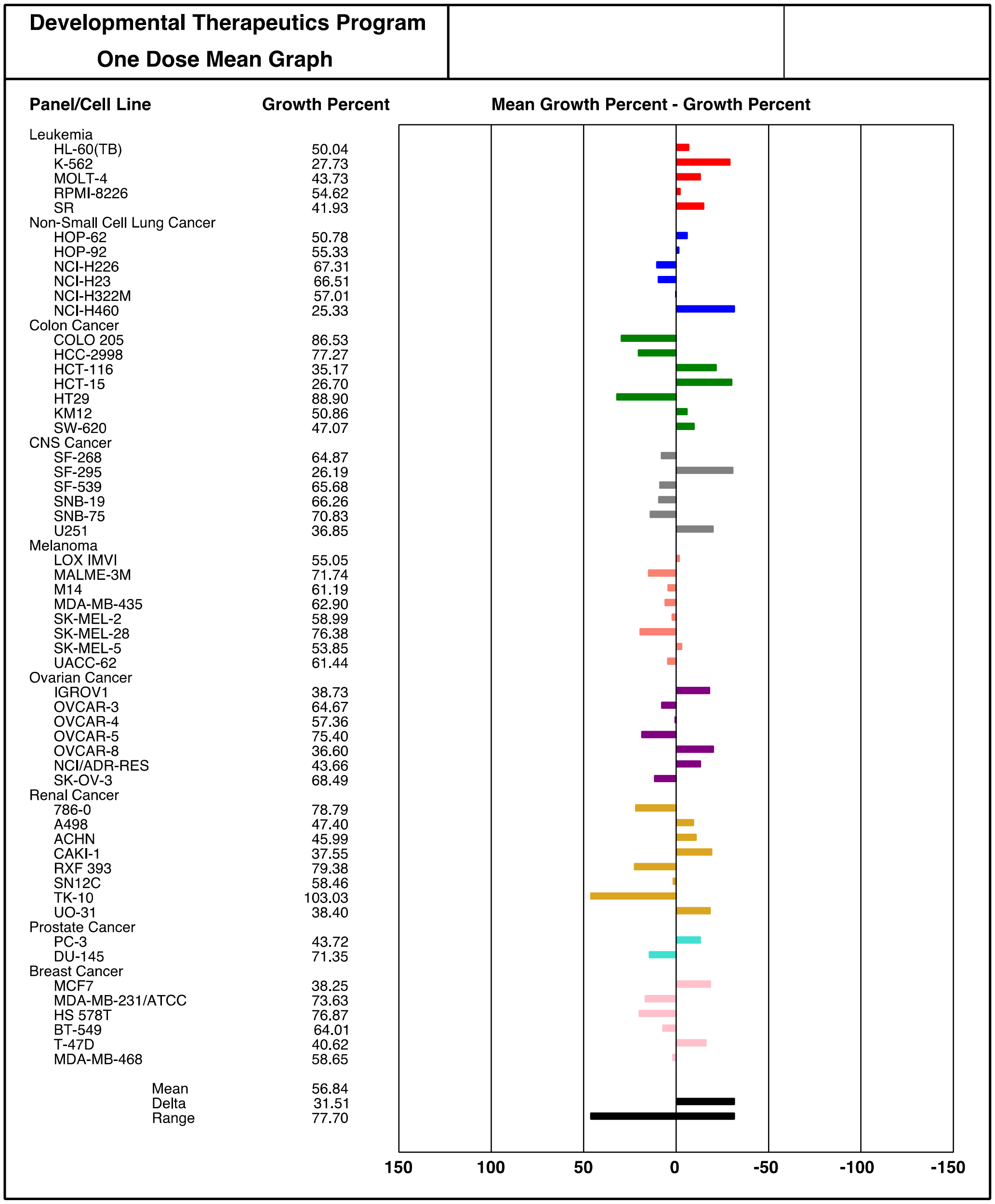

Supplementary Figure 12. NCI 60 cell screening. N106 submitted to the NCI 60 cell screen has been tested initially at a single high dose of 10^{-5} M in 60 different tumor cell panels. 48 hours after compound treatment, cell density and proliferation was determined by sulforhodamine B colorimetric assay. The N106 has not shown any significant oncogenic activity in the NCI 60 cell line screening.

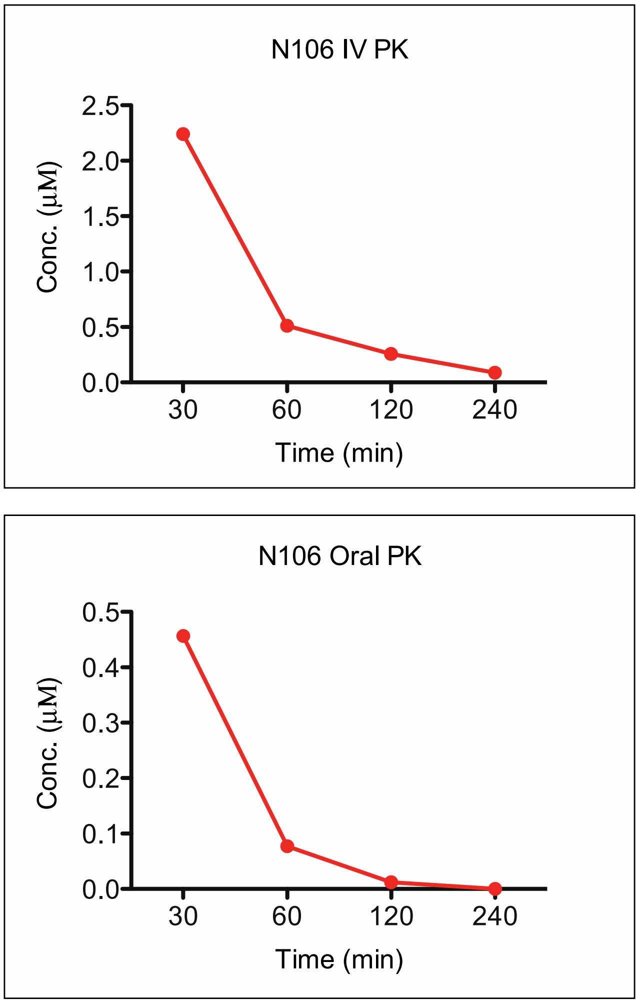

Half Life = 65.4 min
Cmax = 2.24 µM

T 1/2 = 19 min
Cmax = 457 nM
Tmax = 30 min
Oral bioavailability (F%)=56%

Supplementary Figure 13. Pharmacokinetic profile of N106 following administration to mice. The murine PK profile of single dose of N106 (10 mg·kg^{-1}) was determined following intravenously (IV) and oral (PO) administration and analysis of plasma samples was performed by high performance liquid chromatography. Data represents mean value of N106 plasma concentrations in mice. *n* = 3 mice per each time point. Cmax, maximum concentration; T ½, half time; Tmax, time to peak concentration.

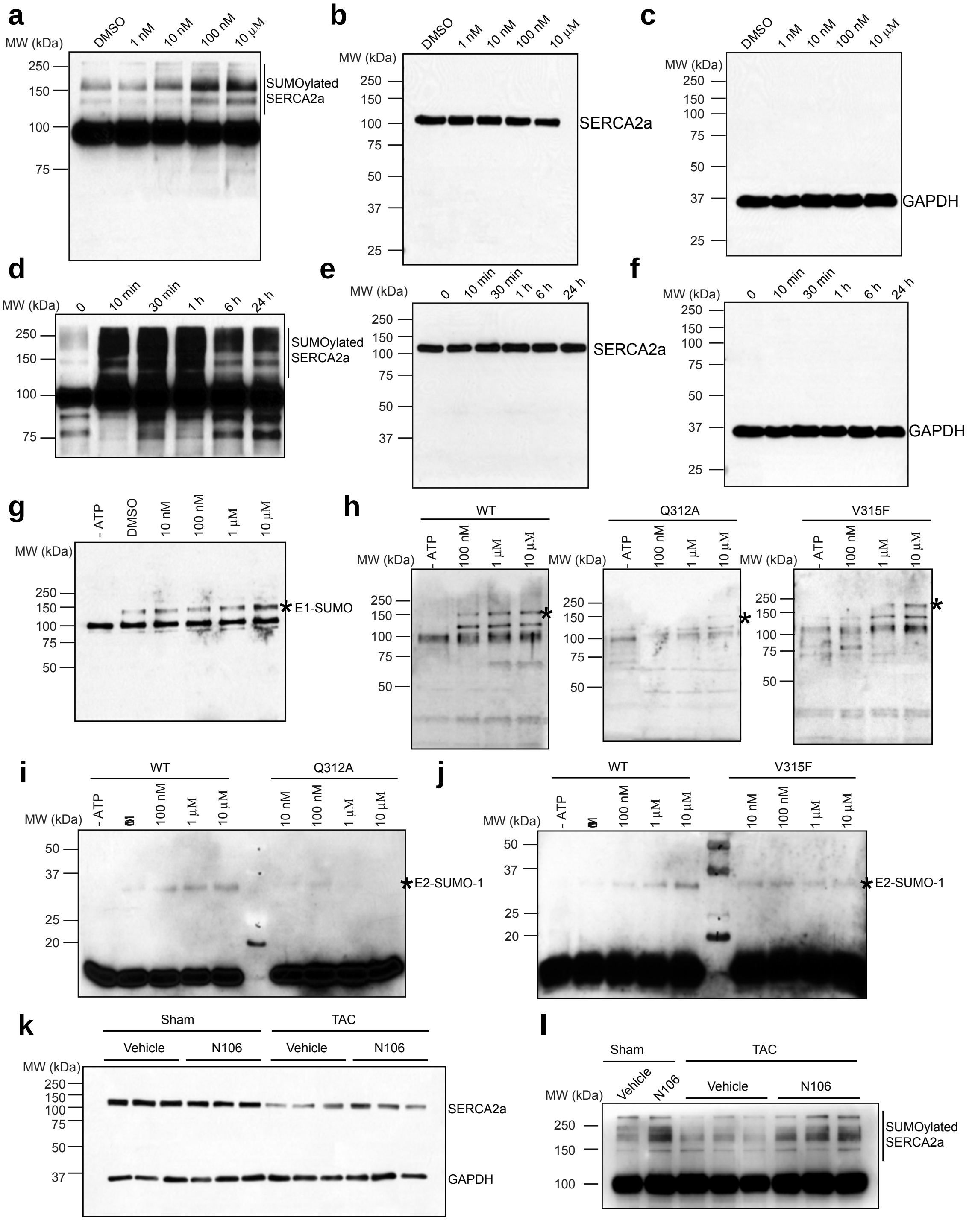

Supplementary Figure 14. Uncropped scanned images of key western blots. uncropped blots used in Figure 1 **(a-c)**, in Figure 2 (**d-f**), in Figure 3 (**g-j**) and in Figure 5 (**k and l**).
